# MicroRNAs and their isomiRs function cooperatively to target common biological pathways

**DOI:** 10.1186/gb-2011-12-12-r126

**Published:** 2011-12-30

**Authors:** Nicole Cloonan, Shivangi Wani, Qinying Xu, Jian Gu, Kristi Lea, Sheila Heater, Catalin Barbacioru, Anita L Steptoe, Hilary C Martin, Ehsan Nourbakhsh, Keerthana Krishnan, Brooke Gardiner, Xiaohui Wang, Katia Nones, Jason A Steen, Nicholas A Matigian, David L Wood, Karin S Kassahn, Nic Waddell, Jill Shepherd, Clarence Lee, Jeff Ichikawa, Kevin McKernan, Kelli Bramlett, Scott Kuersten, Sean M Grimmond

**Affiliations:** 1 Queensland Centre for Medical Genomics, Institute for Molecular Bioscience, The University of Queensland, St Lucia, Queensland, 4072, Australia; 2 Life Technologies, 2130 Woodward St, Austin, TX 78744, USA; 3 Life Technologies, 800 Lincoln Centre Dr., Foster City, CA 94404, USA; 4 Life Technologies, 500 Cummings Center, Suite 2400, Beverly, MA 01915, USA; 5 Epicentre (An Illumina Company), 726 Post Rd, Madison, WI 53713, USA

## Abstract

**Background:**

Variants of microRNAs (miRNAs), called isomiRs, are commonly reported in deep-sequencing studies; however, the functional significance of these variants remains controversial. Observational studies show that isomiR patterns are non-random, hinting that these molecules could be regulated and therefore functional, although no conclusive biological role has been demonstrated for these molecules.

**Results:**

To assess the biological relevance of isomiRs, we have performed ultra-deep miRNA-seq on ten adult human tissues, and created an analysis pipeline called miRNA-MATE to align, annotate, and analyze miRNAs and their isomiRs. We find that isomiRs share sequence and expression characteristics with canonical miRNAs, and are generally strongly correlated with canonical miRNA expression. A large proportion of isomiRs potentially derive from AGO2 cleavage independent of Dicer. We isolated polyribosome-associated mRNA, captured the mRNA-bound miRNAs, and found that isomiRs and canonical miRNAs are equally associated with translational machinery. Finally, we transfected cells with biotinylated RNA duplexes encoding isomiRs or their canonical counterparts and directly assayed their mRNA targets. These studies allow us to experimentally determine genome-wide mRNA targets, and these experiments showed substantial overlap in functional mRNA networks suppressed by both canonical miRNAs and their isomiRs.

**Conclusions:**

Together, these results find isomiRs to be biologically relevant and functionally cooperative partners of canonical miRNAs that act coordinately to target pathways of functionally related genes. This work exposes the complexity of the miRNA-transcriptome, and helps explain a major miRNA paradox: how specific regulation of biological processes can occur when the specificity of miRNA targeting is mediated by only 6 to 11 nucleotides.

## Background

MicroRNAs (miRNAs) are an important class of non-coding regulatory RNAs, which interfere with the translation of protein-coding mRNA transcripts. By incorporation into the RNA induced silencing complex (RISC), miRNAs can inhibit translation [1-6], promote sequestration of mRNAs to P-bodies [7], and/or destabilize and degrade target mRNAs [8-10]. Accumulating evidence suggests that miRNAs are required for achieving precise biological outcomes, such as developmental programs [11-14], and that dysregulation of miRNA expression can drive tumorigenesis and other human pathologies [15-17].

Due to their biological importance, intensive research has focused on understanding the biogenesis of miRNAs (reviewed in [18]). Precursor-miRNA hairpins (pre-miRNAs; approximately 60 to 110 nucleotides) are usually generated in one of two ways: either from the action of Drosha (an RNAse III enzyme) [19,20] on independent primary-miRNA (pri-miRNA) genes; or from Spliceosome-mediated processing of mRNA introns [21,22]. The pre-miRNAs are exported from the nucleus to the cytoplasm [23], where Dicer (another RNAse III enzyme) cleaves the hairpin to produce a miRNA duplex [24,25]. One strand of this duplex is incorporated into RISC [24,26], becoming the mature miRNA (approximately 22 nucleotides). The other strand is often referred to as the miRNA* (miRNA-star), and is often thought to be non-functional and degraded [18,27], although some miRNA* products can be loaded into RISC [28-30]. Recently, an alternative biogenesis pathway was reported for the human miR-451, by which argonaut 2 (AGO2) cleaves the pre-miRNA to generate an intermediate molecule [31], the AGO2-cleaved pre-miRNA (ac-pre-miRNA). The ac-pre-miRNAs are processed to mature miRNAs by exonucleolytic trimming [32,33]. It remains to be seen what proportion of miRNAs are capable of processing via this alternative pathway.

The small size of mature miRNAs (typically only 20 to 24 nucleotides) makes them ideal for characterization using short-tag RNA-sequencing (RNA-seq) technologies [34,35]. Unlike hybridization approaches such as microarray profiling or Northern blotting, massive-scale sequencing provides a way to discriminate discrete but closely related RNA molecules, and profile miRNAs without *a priori *knowledge of expression [36]. RNA-seq has been used to study the miRNA content of a wide variety of species, tissues, and pathologies, with the striking and unexpected observation that pre-miRNAs almost always give rise to more than one mature miRNA sequence regardless of the sequencing platform used [37-46]. These miRNA variants have been dubbed 'isomiRs' [37], and can encompass substitutions, insertions or deletions, 3' end non-templated additions, and 5' and/or 3' cleavage variations.

Despite their consistent appearance in datasets, the biological relevance of isomiRs remains controversial. IsomiRs are commonly dismissed as sequencing artifacts [38,39], alignment artifacts [40], poor quality or degraded RNA [41], sloppy Drosha/Dicer excision [42,43], or simply as 'trivial variants' [44], although some argue that measurement noise cannot account for the high frequency of these variants [45,46]. Recent analyses suggest that some isomiRs may be non-randomly distributed [47,48], which hints that isomiR biogenesis could be regulated and therefore perhaps functional. These observational studies, however, do not suggest possible functions for isomiRs, and they are still potentially confounded by the high error rates of massive-scale sequencing.

Although miRNA populations are thought to be relatively noncomplex (there are only 1,100 human miRNAs annotated in miRBase v15 [49]), the high level of error in massive-scale sequencing combined with the number of possible variants requires ultra-deep sequencing to ensure that isomiRs and poorly expressed miRNAs are detected reliably. Additionally, to understand the biological relevance of isomiRs, multiple biological states from multiple individuals need to be surveyed. In this study we seek to understand the biological relevance of isomiR expression by using ultra-deep miRNA-seq of ten adult human tissues from multiple individuals. We take a deliberately conservative approach to the detection of biologically relevant isomiRs, and validate their association with translating mRNAs. We also use biotin-labeled miRNAs and isomiRs to pull-down endogenous mRNA targets, finding that isomiRs act cooperatively with canonical miRNAs to target common biological pathways.

## Results

### Ultra-deep sequencing and alignment of miRNA-seq tags using miRNA-MATE

In order to thoroughly survey human miRNA expression, we constructed 30 libraries from 11 individual donors across 10 adult human tissues (brain, heart, kidney, liver, lung, ovary, placenta, spleen, testes, and thymus), and sequenced them using the SOLiD (Life Technologies) sequencing platform (Table 1; Table S1 in Additional file 1). As mature miRNAs are small, they contain limited information that can be used to place them uniquely in the genome (Figure S1 and Table S2 in Additional file 1). To maximize the accuracy of miRNA mapping, four parameters need to be optimized: (i) the appropriate choice of reference sequence; (ii) the number of mismatches allowed during alignment; (iii) the minimum length of tags to be aligned; and (iv) careful and conservative filtering of biological signal from noise.

**Table 1:** Sequencing statistics for the small RNA tissue panel

Tissue	Number of libraries	Number of runs	Number of individuals	Mature mapped tags (recursive)	Mature mapped tags (adaptor trimming)	Hairpin mapped tags (recursive)	Hairpin mapped tags (adaptor trimming)
Brain	1	2	1	3,967,251	1,241,131	5,582,668	1,321,577

Heart	3	4	1	28,090,024	29,306,067	44,738,409	29,684,452

Kidney	3	4	2	33,358,423	34,126,218	50,899,691	38,556,827

Liver	3	4	2	20,138,397	20,067,003	30,613,223	23,032,850

Lung	5	6	1	48,640,701	45,419,062	69,465,607	49,924,703

Ovary	4	4	1	33,291,714	34,240,510	61,752,617	46,059,416

Placenta	2	2	1	24,557,859	19,582,092	32,597,796	18,765,252

Spleen	3	4	1	28,616,021	30,285,592	42,144,297	32,390,566

Testes	3	4	2	21,538,067	22,458,615	35,716,355	28,385,304

Thymus	3	4	2	40,180,905	39,883,625	54,627,498	43,278,516

To address these issues, we have created a miRNA-seq analysis pipeline ('miRNA-MATE'), which analyses SOLiD miRNA-seq data using two different alignment strategies (Figure S2 in Additional file 1). The first strategy, dubbed 'recursive mapping' [50], attempts to align sequences at their longest length, iteratively trimming and re-aligning if unsuccessful. This approach provides the best sensitivity for quantifying miRNA expression, but cannot determine the precise end of the captured miRNA. The second strategy ('adaptor trimming') was optimized for studying isomiRs, where knowing the exact length of the captured tag is crucial. For a full description of miRNA-MATE and mapping parameters used, see the Materials and methods, and Additional file 2.

In total, almost 430 million tags could be recursively aligned to miRNA hairpins represented in miRBase v15 [49], of which approximately 73% could also be precisely identified via the adaptor trimming approach (Table 1). To set a conservative threshold of genuine expression, we also aligned to an optimized reference library containing the mature miRNAs from all available species in miRBase v15 (Table 1). To examine cross-species mapping, we attempted to align all non-human, non-conserved miRNAs to human pre-miRNA hairpins without success (data not shown). Therefore, we would not expect short tags derived from a human library to align optimally to mature plant miRNAs unless they were generated by random error (for example, multiple templates on a single bead, contaminating signal from nearby beads or other causes). We could therefore use these non-human, non-conserved miRNAs to set a minimum threshold for genuine expression. In this set of libraries, miRNAs were considered as expressed if they were detected at ≥ 10 transcripts per million (tpm) (Figure S3 in Additional file 1), and across all tissues we identified expression from 470 mature miRNAs from 423 hairpins (Table S3 in Additional file 1). Although this is a very conservative estimate of the noise level, this still allowed us to query the biological relevance of isomiRs with the open bias that highly expressed sequences are more likely to be biologically relevant. Hierarchical clustering of the miRNAs expressed above this threshold shows profiles clustering by tissue, confirming that the threshold was sufficiently conservative (Figure S4 in Additional file 1).

### Confirmation of sequencing data with quantitative RT-PCR and microarray analyses

**Figure 1:**
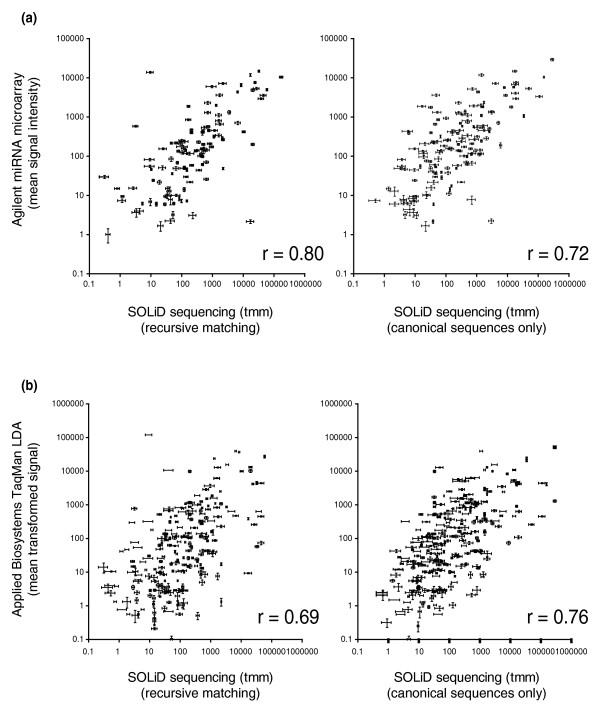
**Correlation between miRNA-seq, qRT-PCR, and microarrays for placental miRNAs**. Different alignment strategies will produce different correlations due to the characteristics of the validation technology. **(a) **The Agilent miRNA microarray typically will capture signal from isomiRs due to cross-hybridization, and therefore has a higher correlation to the recursive matching strategy, which identifies both miRNAs and isomiRs. **(b) **TaqMan LDA qRT-PCR is more sensitive to the exact sequence of the miRNA, and therefore has higher correlations to the adaptor trimming strategy, which was filtered for exact matches to canonical miRNA sequences. In all plots, points represent mean detection values, and bi-directional error bars represent standard error. ρ values indicated are Spearman correlations. These comparisons demonstrate that the miRNA-seq technology and our analysis approach are both sensitive and specific.

Quantitative RT-PCR (qRT-PCR) is often considered to be the gold standard for analysis of gene expression data. To confirm our sequencing results, and assess the accuracy and sensitivity of the different alignment strategies, we analyzed miRNA expression from the same RNA samples using TaqMan low density qRT-PCR arrays. Additionally, for one tissue (placenta) we also analyzed expression by Agilent miRNA microarray. These two validation strategies capture different attributes of the RNA sample. Agilent miRNA microarrays will capture signal from canonical miRNAs as well as isomiRs due to cross-hybridization with probes designed for canonical miRNAs. In contrast, TaqMan LDA qRT-PCR is highly sequence-specific for canonical miRNAs. Figure 1 emphasizes this point, showing that the Spearman rank correlations for microarray and qRT-PCR data are best for recursive (ρ = 0.80) and canonical sequence-specific analyses (ρ = 0.76; Figures S5 and S6 in Additional file 1), respectively. Both microarray and qRT-PCR show high concordance with the sequencing data, demonstrating our analysis is both sensitive and specific.

### A standard nomenclature for isomiR description

**Figure 2:**
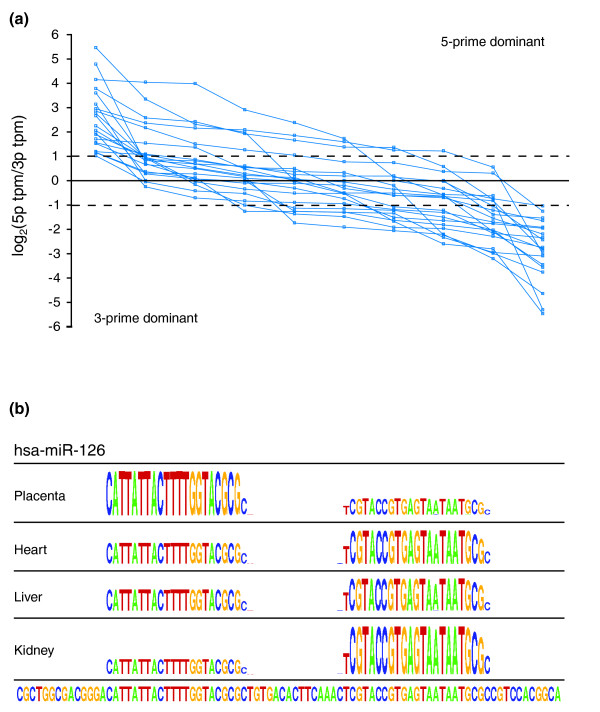
**Arm switching frequency in the miRNA-seq data**. **(a) **Arm-specific expression of at least 21 miRNAs varied between tissues. For all 10 tissues (points), log ratios of 5p to 3p arm expression for 21 miRNAs (blue lines) are plotted according to the arm on which dominant expression occurred, clearly illustrating differences between the tissues for each miRNA. **(b) **Sequence logo plots for hsa-miR-126 showing the switch between expression dominance from the 5p arm (placenta) to the 3p arm (heart, liver, and kidney). The pre-miRNA hairpin is displayed along the bottom.

Although the existing 'miRNA/miRNA-star' annotations give instant functional context, the major disadvantage when used as a nomenclature is that these annotations are not based on static characteristics of the miRNA - the dominant strand could change in different biological settings leading to different names describing the same molecule. To investigate the scope of this problem, we examined how often the dominant arm of the pre-miRNA hairpin switched across different tissues. Of 163 hairpins with expression of ≥ 10 tpm in both the 5p and 3p arms, we identified 21 (12.9%) whose dominant expression switched from one arm to the other in at least one tissue (Figure 2; Table S4 in Additional file 1). Considering that the tissues sampled here represent only a small fraction of known biological diversity, this figure represents a lower rather than upper bound for arm-switching. In this context, the star-based nomenclature is not-informative, and we (along with miRBase curators) argue that this nomenclature should be abandoned in favor of the '5p' and '3p' annotations.

We propose a machine parsable nomenclature based on the sequence of the isomiR and the relative positions in the pre-miRNA hairpin. The details are presented in Additional file 3, but as an example: the notation 'hsa-miR-143-3p|{hsa-miR-143}|60_80|' refers to the canonical sequence of hsa-miR-143-3p (annotated in miRBase as hsa-miR-143); and 'hsa-miR-143-3p|{isomiR}|62_80|sub.77.G > A' refers to an isomiR of hsa-miR-143 that starts two nucleotides downstream of the canonical sequence, and contains a substitution from G to A at position 77 in the hairpin. Much of this nomenclature has been based on existing strategies for the description of mutations in nucleotide data, and should therefore be reasonably intuitive in its application to miRNAs. We have used this nomenclature for the remainder of this study. A tab delimited text file containing all detected isomiRs and their counts in each sample can be found in Additional file 4.

### Classification and characterization of isomiRs

**Figure 3:**
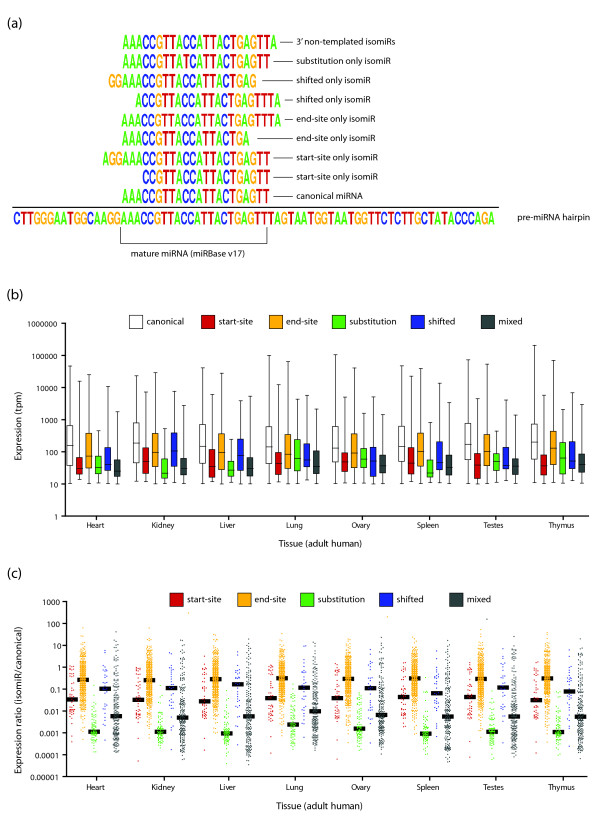
**miRNA and isomiR characteristics**. **(a) **Example of a canonical miRNA and each mutually exclusive isomiR category. IsomiRs that would fit more than one of these categories are classified as 'mixed' isomiRs. **(b) **Box-whisker plots showing expression of isomiR types within each tissue. **(c) **Ratio of each isomiR to its canonical miRNA grouped according to isomiR type within each tissue.

Having annotated our ultra-deep sequencing data with isomiR-specific nomenclature, we then sought to understand some of the basic characteristics of isomiRs and compared them to the characteristics of canonical miRNAs. We first classified the tags into seven mutually exclusive categories: (i) canonical miRNAs; (ii) start-site-only isomiRs; (iii) end-site-only isomiRs; (iv) substitution-only isomiRs; (v) shifted isomiRs (those isomiRs where the length is identical to the canonical sequence, but there are variations in both the start and end sites); (vi) 3' non-templated addition isomiRs (isomiRs where additional nucleotides are present at the 3' end that do not match the reference hairpin sequence); and (vii) the remaining 'mixed' isomiRs, which are typically combinations of the prior categories (Figure 3a). To ensure the robustness of our results, we required that an isomiR be present at a minimum of 10 tpm in every sample of at least one tissue, and that the isomiR must have been detected by at least two different library preparation methods. This effectively excluded isomiRs that were present only in the brain and placenta, and isomiRs that were specific to an individual donor (Table S1 in Additional file 1). We also excluded 'orphan' isomiRs (isomiRs for which the canonical miRNA sequence was not detected (Table S5 in Additional file 1)), as these could possibly arise from mis-annotated mature miRNAs. Applying these additional filters, we detected 190 canonical miRNAs and 834 isomiRs. As we did not detect any 3' non-templated addition isomiRs above the expression threshold in any tissue, this class of miRNA was not considered further in this study.

We then sought to examine the expression characteristics across each of these categories (Figure 3b). In most tissues, start-site isomiRs, substitution isomiRs, and mixed isomiRs show greatly reduced expression compared to the canonical miRNAs, whereas end-site isomiRs and shifted isomiRs both show either comparable or slightly reduced expression. We hypothesized that the limited expression of substitution-only isomiRs was predominantly due to sequencing errors, and that the expression ratios of these isomiRs would be very low compared to the corresponding canonical miRNA. Typically, the median ratio for substitution-only isomiR tpm/canonical miRNA tpm was around 1/1,000, and there were very few instances of this ratio exceeding 1/10, suggesting that this category cannot be distinguished from sequencing artifacts (Figure 3c). In contrast, the median ratio for end-site-only isomiRs was approximately 1/3, and there were a great many with ratios exceeding 1, indicating that these isomiRs were more abundant than their canonical miRNAs. Start-site-only and shifted isomiRs also have a greatly increased median compared to substitution-only isomiRs: approximately 1/30 and approximately 1/10, respectively. Together, these results suggest that most categories of isomiRs are unlikely to be merely artifacts of sequencing or library generation.

One isomiR feature that has been identified in previous studies but not confirmed here was the over-abundance of U insertions when compared to U deletions [51]. We find no substantial evidence for widespread insertion or deletion events in any tissue, identifying at most 0.54% of input tags as containing deletions, and 0.066% of tags containing insertions (Figure S7 in Additional file 1). This is likely due to the sequencing platforms used: in the Reid *et al*. study [51] the authors utilize 454 and Illumina sequencing, both of which are more susceptible to insertion or deletion artifacts than SOLiD sequencing used here. Such biases in miRNA detection have been seen previously [52], and highlight that the exact protocols to capture and measure isomiRs will influence the results, and should be considered when interpreting the relative importance of either individual isomiR sequences, or broad isomiR classes.

### The ac-pre-miRNA pathway contributes substantially to end-site only isomiRs

**Figure 4:**
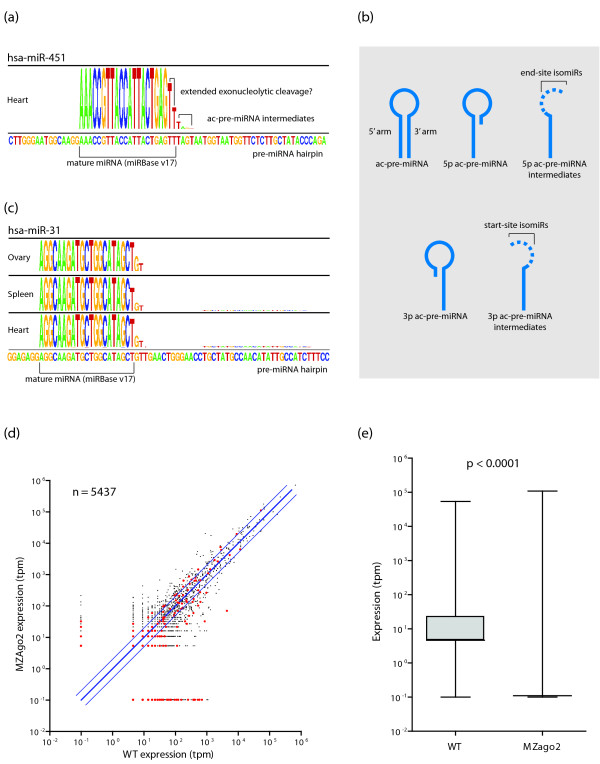
**Identification of ac-pre-miRNA candidates**. **(a) **Sequence logo profile of hsa-miR-451 showing 5' arm dominance, almost no variation in the 3' end of the mature miRNA, and a trail of 3' isomiR extensions. **(b) **Schematic diagram illustrating the generation of end-site (5p-ac-pre-miRNA) and start-site (3p-ac-pre-miRNA) isomiRs via AGO2-mediated cleavage of a pre-miRNA. Dashed lines indicate variable regions. **(c) **Sequence logo profile for hsa-miR-31, which was identified by our analysis as a candidate for biogenesis through the ac-pre-miRNA pathway. Three representative tissues are shown. **(d) **Wild-type (WT) zebrafish whole embryo versus mutant AGO2 inactive (MZAgo2) whole embryo miRNA and isomiR expression. Each point represents a separate isomiR or miRNA. The thick blue line is the perfect correlation line, the thin blue lines are ± 2-fold visual guides. Red dots indicate isomiRs/miRNAs derived from candidate ac-pre-miRNA hairpins. A bias towards down-regulation in AGO2 mutants is evident in this plot. **(e) **Box and whiskers plots showing the expression of all isomiRs/miRNAs deriving from ac-pre-miRNA candidates in wild-type (WT) and mutant (MZago2) miRNA-seq samples; 75% of ac-pre-miRNA candidate molecules are not expressed in the mutant sample.

The recent discovery of an alternative miRNA biogenesis pathway potentially explains the presence of a large proportion of the isomiR population. AGO2-mediated cleavage of the pre-miRNA produces intermediates (ac-pre-miRNA) that are detectable by miRNA-seq. We examined the sequence logos from the hsa-miR-451 pre-miRNA, known to participate in this pathway [32,33], observing three major characteristics: (i) the consistency of the 5' end of the mature sequence; (ii) the dominance of the 5' arm (while the 3' arm can sometimes be detected, it is not observed above 10 tpm in any tissue); and (iii) the substantial tail of ac-pre-miRNA intermediates (Figure 4a). To understand the scope of ac-pre-miRNAs within the isomiR population, we searched for hairpins displaying these expression characteristics on either arm of the hairpin (Figure 4b), restricting our analysis to the eight tissues with multiple library preparation types (heart, kidney, liver, lung, ovary, spleen, testes, and thymus). In this way, we identified an additional 46 5p-ac-pre-miRNA candidates (Figure 4c; Table S6 in Additional file 1), but did not find evidence of 3p-ac-pre-miRNAs, perhaps reflecting the relative activity of 5' to 3' RNA exonucleases compared to 3' to 5' exonucleases [53]. Together, these potential ac-pre-miRNA intermediates account for 62.8% of the end-site isomiR expression, and 48.3% of all isomiR expression observed in this study.

To confirm the association of these ac-pre-miRNA candidates with AGO2-mediated cleavage, we examined the miRNA-seq profiles of wild-type zebrafish whole embryos, and mutant embryos where AGO2 was catalytically inactive [33]. Using miRNA-MATE to align these data to *Danio rerio *hairpins (miRBase v17), we identified 13 ac-pre-miRNA candidates with expression of the 5p canonical miRNA in the wild-type sample. As a group, the miRNAs and isomiRs deriving from the ac-pre-miRNA candidate hairpins have significantly lower expression in the AGO2 mutants (Figure 4d, e; Chi-square value = 12021.776; 1 degree of freedom; *P *< 0.0001). Of the 13 candidates, 9 had complete ablation of canonical expression with inactive AGO2, and an additional 3 had a reduction in expression of > 80%. IsomiR expression patterns also support all but one of these 12 miRNAs, with expression < 80% of wild-type levels, leaving 11/13 (84.6%) candidates with strong experimental support for AGO2 cleavage (Table S6 in Additional file 1). These data support a more extensive role for AGO2-mediated cleavage in miRNA/isomiR biogenesis than has previously been appreciated.

### IsomiRs are actively associated with the transcriptional machinery

**Figure 5:**
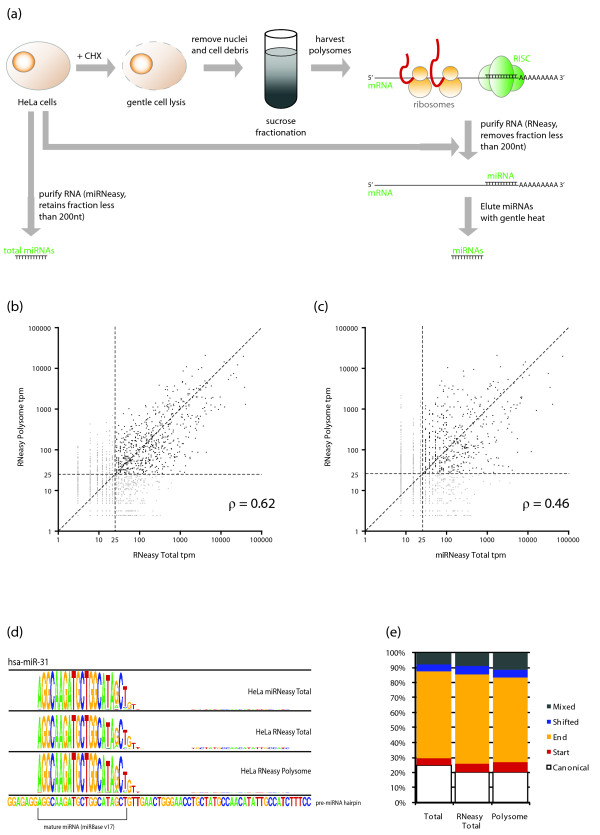
**IsomiRs are associated with translational machinery**. **(a) **Schematic diagram showing the laboratory workflow to isolate total miRNA, miRNA associated with mRNA, and miRNAs associated with polysomal mRNA. **(b, c) **Correlation scatter plots describing miRNA expression in the three preparations described in (a). The noise threshold was set at 25 tpm for these libraries. (b) Correlation scatter plot between miRNA associated with mRNA (x-axis), and miRNA associated with polysomal mRNA (y-axis). The dashed diagonal line represents perfect correlation, while the horizontal and vertical lines represent the likely noise thresholds for these data. (c) Correlation scatter plot between total cellular miRNAs, and polysomal associated miRNA. The dashed diagonal line represents perfect correlation, while the horizontal and vertical lines represent the likely noise thresholds for these data. **(d) **A sequence logo profile for hsa-miR-31 showing similar profiles for all three HeLa miRNA-seq samples. This example shows an ac-pre-miRNA candidate hairpin with no change in the proportion of intermediates in the translational machinery. **(e) **Proportional bar graph illustrating the similar distribution of isomiR categories in each of the three HeLa miRNA-seq samples. CHX, cycloheximide, nt, nucleotide.

Previous studies have identified isomiRs associated with the AGO proteins [45,54,55], suggesting that these molecules can incorporate into endogenous RISC. We sought to further clarify the biological relevance of isomiRs by determining whether these isomiR-containing RISC complexes were capable of binding to endogenous mRNAs. As the RISC complex is associated with translating mRNA (polysomes) [4,56], we purified polysomal RNA from HeLa cells using a sucrose gradient fractionation (Figure S8A in Additional file 1) followed by a standard column and ethanol concentration, designed to capture RNAs longer than 200 nucleotides, thus discarding any miRNAs not bound to long polysomal mRNA (Figure 5a). We then eluted the bound miRNAs using a gentle heating step, and prepared this miRNA fraction for sequencing (Figure 5a). We also prepared total cell miRNAs, and total cell RNA-bound miRNAs.

The quality of all RNA preparations was checked, revealing no evidence of degradation (Figure S8B in Additional file 1). We confirmed that the RNeasy columns did not retain miRNAs in the absence of longer mRNAs by qRT-PCR of HeLa RNA processed in five ways: (1) RNeasy column (to confirm detection of miRNAs); (2) RNeasy column-purified RNA that has been heat denatured and re-purified on an RNeasy column (to remove the bound miRNAs); (3) RNeasy column-purified RNA that has been re-purified on an RNeasy column (to confirm that miRNAs are stable after multiple purifications); (4) miRNeasy column (to confirm detection of miRNAs); and (5) miRNeasy column-purified RNA that has been heat denatured and re-purified on a miRNeasy column (to confirm that miRNAs can still be detected after heat denaturation). The selective loss of miRNA detection after heat denaturation and RNeasy purification, but not heat denaturation and miRNeasy purification, or double RNeasy purification confirms that miRNAs are stably associated with longer RNAs, and are not retained on the RNeasy column in the absence of longer RNAs (Figure S8C in Additional file 1). We also checked for the enrichment of polysomes in our sample preparations by assessing the miRNA-seq libraries for: (i) ribosomal RNA (expected to be enriched in the polysome fraction relative to mRNA associated fraction); (ii) tRNA (expected to be moderately depleted in the polysome fraction relative to the total RNA fraction); and (iii) small nucleolar RNA (snoRNA; expected to be greatly depleted in the polysome fraction). We found a 3.9-fold enrichment of rRNAs, an 8.1-fold depletion of tRNAs, and > 50-fold depletion of snoRNAs in polysome RNAs longer than 200 nucleotides when compared to total RNAs longer than 200 nucleotides. These data confirm that the polysome fraction is of sufficient purity to analyze miRNAs associated with actively translating ribosomes.

As expected, the expression levels of polysome-associated miRNAs were well correlated (Spearman ρ = 0.62) with those of total mRNA-bound miRNAs (Figure 5b), confirming the dominant function for cytoplasmic RNA-bound miRNAs is to bind to mRNAs. The correlation between polysome miRNAs and total HeLa miRNAs was far less (Spearman ρ = 0.46; Figure 5c), suggesting that there are a number of miRNAs that are not localized to the cytoplasm, likely reflecting the other non-targeting functions of miRNA [57]. The repertoire of isomiRs identified in the polysome miRNA fraction was similar to that found in the total RNA-bound fraction. Approximately 97% of isomiRs detected in the total RNA-bound fraction were also detected in the polysome fraction, and despite a polysome enrichment of at least four-fold, the distribution of isomiRs in these two fractions was essentially identical (Figure 5d, e). This demonstrates that isomiRs of all categories are associated with the translational machinery, and thus are highly likely to be functional in mammalian cells. We also compared the proportion of isomiRs from the ten adult human tissues, and checked for their expression in the HeLa polysome-associated fraction. Except for substitution only, and mixed isomiRs (which contain a large proportion of nucleotide changes), approximately 30% of the tissue atlas isomiRs in each category were also detected in the HeLa polysome fraction (Table S7 in Additional file 1), again confirming the relevance of these particular isomiR types.

### IsomiR expression is highly correlated with canonical miRNA expression

Having determined that isomiRs are expressed at biologically meaningful levels, and that they are associated with the translational machinery, we then investigated the biological role of isomiRs and their relationship to canonical miRNAs. We hypothesized that if isomiRs were highly correlated with canonical miRNAs, they would be likely to drive similar biology. Equally, if they were not highly correlated, they would drive different biology. Across all categories of isomiRs, we found isomiRs to be highly correlated with canonical miRNA expression (Figure S9 in Additional file 1). Only small differences were observed between categories, with the median Pearson correlation ranging from approximately 0.9 for shifted isomiRs to approximately 0.96 for end-site isomiRs. In addition, more than 75% of isomiRs had Pearson correlations of > 0.75 with their canonical miRNAs. Whilst we acknowledge that the small differences observed could potentially represent differences in isomiR biogenesis and regulation, we conclude that most isomiRs are likely to be driving similar biology to their canonical miRNAs.

### IsomiRs increase the specificity of miRNA targeting

**Figure 6:**
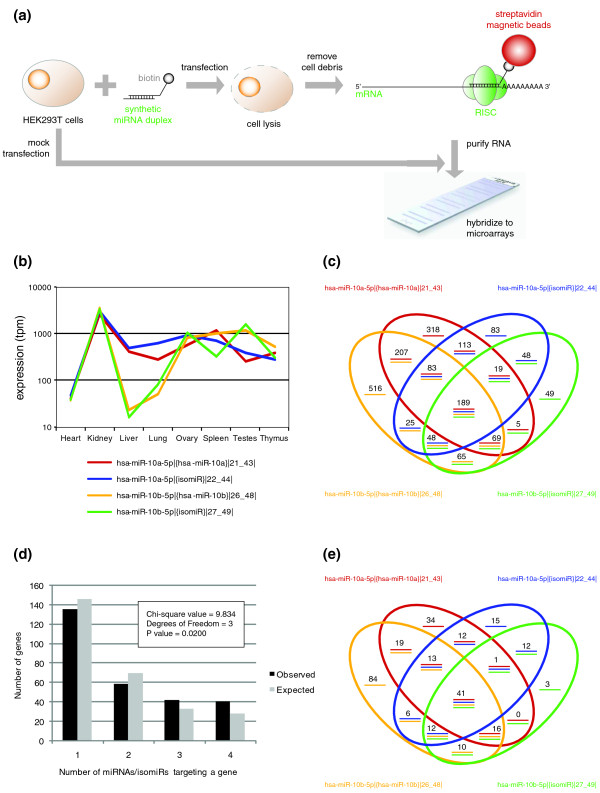
**IsomiRs target similar molecules to their canonical miRNAs**. **(a) **Schematic diagram depicting the laboratory workflow for the pull-down of mRNA targets of a synthetic biotinylated miRNA. **(b) **The miRNA expression profiles of the two miRNAs and corresponding shifted isomiRs selected for pull-down experiments. **(c) **A four-way Venn diagram depicting the overlap between the target genes detected by microarray analysis of pulled-down fractions. **(d) **The distribution of the number of miRNAs/isomiRs that target genes from significantly enriched pathways. There are more genes targeted by three or more isomiRs than would be expected by chance, and less genes targeted by two or fewer isomiRs than would be expected by chance. **(e) **A four-way Venn diagram displaying the specific targeting of the genes used in (d).

For miRNAs involved in the translational machinery, their biological role can be understood by the mRNA targets they repress. Target site prediction is generally based on 'seed' site interactions, although miRNAs can bind their targets in many different ways [58]. Additionally, existing prediction algorithms are notorious for false positives [59-61]. To determine the direct mRNA targets of miRNAs and isomiRs in a high-throughput manner, we used biotin-labeled synthetic miRNAs or isomiRs to pull-down endogenous mRNA targets and profiled these fractions by microarray (Figure 6a) [62,63]. We first optimized this technique for miR-17-5p, a miRNA we have previously characterized as a regulator of cell cycle progression [64], and a miRNA for which there are more than 30 mRNA targets validated by reporter gene assay [64-67]. Using a threshold equivalent to a false discovery rate of 5%, we were able to identify 21 of the 34 validated mRNA targets above this threshold (Figure S10A in Additional file 1). Additionally, gene set enrichment analysis (GSEA) of the miR-17-5p pull-down genes corroborates the understood biology of this miRNA (Figure S10B in Additional file 1), confirming the sensitivity and specificity of this assay.

We then selected two closely related miRNAs (hsa-miR-10a-5p|{hsa-miR-10a}|21_43| and hsa-miR-10b-5p|{hsa-miR-10b}|26_48|), and a shifted isomiR for each of these (hsa-miR-10a-5p|{isomiR}|22_44| and hsa-miR-10b-5p|{isomiR}|27_49|), that displayed concordant expression across tissues (Figure 6b). We performed biotin pull-downs for each of these four molecules, independently replicated, and assayed the captured mRNAs by microarray. As expected by target prediction, each pull-down enriched expression of hundreds of mRNAs relative to mock transfection controls. Microarray results showed that between 492 and 1,208 genes were significantly enriched in the pull-downs, many of which were common to several of the four pull-downs (Figure 6c).

**Figure 7:**
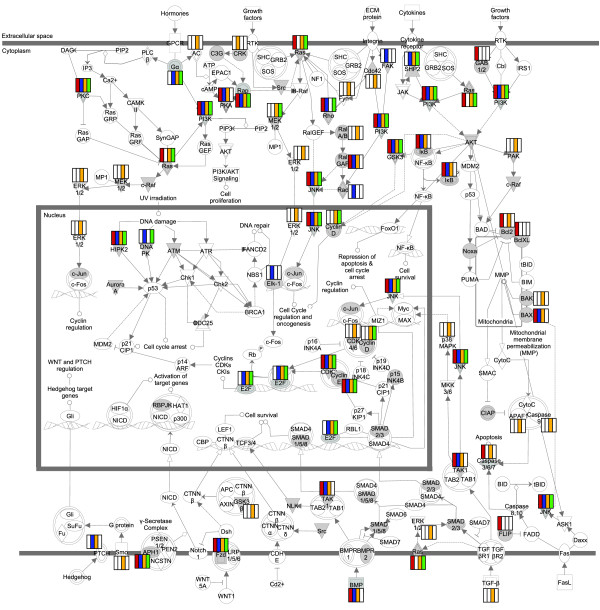
**IsomiRs and miRNAs target similar biological pathways**. The 'molecular mechanisms of cancer' pathway (Ingenuity Pathway Analysis) overlaid with rectangles corresponding to the miRNA or isomiR targeting that gene. hsa-miR-10a-5p|{hsa-miR-10a}|21_43| is colored red, hsa-miR-10b-5p|{hsa-miR-10b}|26_48|) is colored yellow, hsa-miR-10a-5p|{isomiR}|22_44| is colored blue, and hsa-miR-10b-5p|{isomiR}|27_49| is colored green.

The miR-10 miRNAs are often dys-regulated in cancers, and have been shown to act as oncogenes by targeting the HOX genes and other tumor suppressors (reviewed in [68]). In our data set, we found that targets of miR-10 miRNAs and isomiRs were enriched in pathways that are directly involved in cancer (such as cell cycle regulation and apoptosis signaling), and in signaling pathways that contain important cancer molecules. One such pathway, 'the molecular mechanisms of cancer', was the most significant pathway for hsa-miR-10b-5p|{hsa-miR-10b}|26_48| (*P *= 8.32 × 10^-7^), but was also significant for the other three pull-downs (*P *= 2.85 × 10^-3 ^to 9.33 × 10^-5^). Many key genes of this pathway were targeted by multiple miRNAs/isomiRs (including those encoding RAS, PI3K, JNK, E2F, PKC, and HIPK2), but the combination of miRNAs/isomiRs varied from gene to gene (Figure 7). Such a targeting pattern suggests that isomiRs increase the dosage of miRNA-mRNA targeting to core biological processes, whilst distributing the 'off-target' effects randomly through the transcriptome.

**Figure 8:**
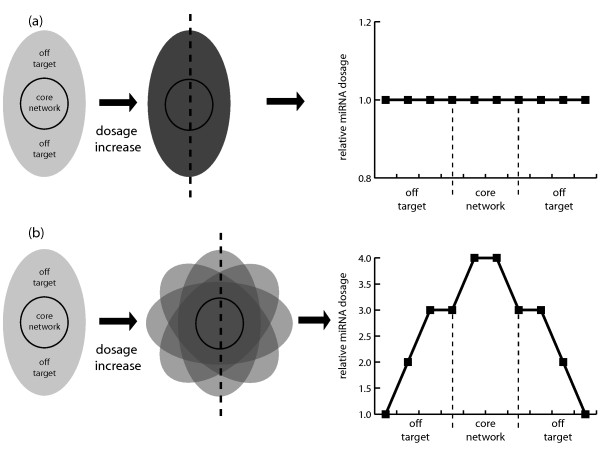
**A diagram illustrating how isomiRs may act cooperatively with the canonical miRNAs to increase the signal to noise ratio of mRNA targeting**. **(a, b) **Where only a single miRNA molecule is used for targeting (a), there is no differentiation between potential 'off-target' effects, and targeting the 'desired' core molecular network when the dosage of that miRNA is increased. However, the dosage is increased using isomiRs (b), with slightly different targeting of both core network mRNAs and 'off-target' miRNAs, amplification of specific targeting of the 'core' network can be achieved relative to the 'off-target' effects. The more isomiRs present, the more differentiation could be achieved.

If hypothesis is true, it would follow that mRNAs targeted by multiple miRNAs/isomiRs would be enriched in functional networks compared to other parts of the transcriptome. To test this, we compared the repertoire of significantly enriched pathways identified when filtering the gene lists by the number of miRNAs/isomiRs that target these genes. Of the 44 significantly enriched pathways (*P *≤ 0.01) identified by at least two pull-down experiments, we were able to identify 25 (56.8%) and 29 (65.9%) pathways when filtering for genes identified by at least two or three pull-downs, respectively. The converse also holds true - we collected all pull-down genes in a given pathway, where that pathway was significantly enriched (*P *≤ 0.01) for at least one member of the miR-10 family (96 pathways; 284 genes). We then asked whether the distribution of miRNA targeting was different for this set of genes than would be expected by the distribution of genes that were pulled-down by any miRNA. We found a statistically significant difference between these two distributions (Chi-square value = 9.834; 3 degrees of freedom; *P *= 0.0200), with an enrichment of genes that were targeted by three or more miRNAs/isomiRs (Figure 6d, e). These results confirm that genes targeted by multiple miRNAs/isomiRs are more likely to be enriched in a functional pathway, and that those that are targeted by less miRNAs/isomiRs are likely to be off-target interactions that are distant from the miRNA's central biology. When taken together, all these results support the hypothesis that miRNAs and their isomiRs act cooperatively to drive similar biology, and that the generation of isomiRs could increase both the signal to noise ratio and the potential dynamic range of miRNA effect (Figure 8).

## Discussion

Almost every study analyzing miRNA-seq results has reported the presence of isomiRs, although their biological role has remained unclear. In this study, we have attempted to resolve the controversy by using thoroughly deep and extensively verified sequencing data and alignment strategies, operating under the assumption that more highly expressed sequences are likely to be more biologically relevant. Whilst we do not believe that biological importance correlates with transcriptional levels, we did aim to set conservative thresholds in order to counter the common argument that rare transcripts (particularly those with less than one transcript per cell) are less likely to be essential biological components. Arguably, this filtering may have been over-conservative, removing interesting and relevant biological models. For example, we did not detect 3' non-templated addition isomiRs above our stringent threshold, although this class of isomiR has been previously reported as developmentally regulated in *Drosophila melanogaster *[48].

Despite the aggressive filtering, most categories of isomiRs were detected well above the signal thresholds at comparable levels to canonical miRNA sequences, and their presence in individual donors across multiple tissues argues against these molecules being sequencing artifacts. Supporting the assertion that these molecules are functional, we find that isomiRs are specifically bound to translating mRNAs in the cytoplasm at similar proportions seen in total miRNA profiling (Figure 5e). This confirms that the association of isomiRs with AGO complexes is due to active inhibition of mRNA targets and is not the result of the accumulation of inactive complexes.

Some categories of isomiRs appear to be less relevant than others, and we find that isomiRs that differ only by nucleotide changes are more likely to be artifacts of the library preparation and/or sequencing based on their low absolute expression (Figure 3b), expression relative to their corresponding canonical miRNAs (Figure 3c), and their poor retention in active RISC (Table S7 in Additional file 1). However, these results cannot necessarily be generalized and should not be used to argue that every individual substitution isomiR is an artifact. Even within the substitution isomiRs, there are a few that pass both absolute and relative expression filters, and these could be candidate allelic-specific expression or RNA editing events. It is important to also consider the method of data generation when interpreting the results from sequencing: all enzymatic steps in library preparation include biases that manifest in the relative detection levels of miRNAs (and presumably isomiRs too) [52]. These biases could affect the detection of an individual isomiR relative to its canonical miRNA, or relative to other sequencing platforms, and could also distort the relative importance of isomiR classes. One such example is our finding that isomiR substitution and deletion events are less common than previously reported [51], a difference most likely due to the different sequencing technologies used. On a genome-wide scale the impact of such biases is often lessened, but not removed entirely. Further study to pinpoint the sources of bias in small RNA sequencing will allow the normalization of sequencing results, and a more accurate estimation of the relative abundance of isomiRs.

The depth of sequence used here has allowed us to study the number of miRNAs potentially undergoing alternative biogenesis as their primary method of production. This is an important consideration as almost half of the isomiRs detected in this study may have arisen as the result of ac-pre-miRNA cleavage and exonucleolytic activity. Support for this comes from significant ablation of expression from candidate ac-pre-miRNA hairpins when AGO2 is inactivated (Figure 4); however, cleavage imprecision from Drosha or Dicer may also account for this isomiR biogenesis. This explanation would require that the fidelity of cleavage at the 5' end is greater than at the 3' end. For Dicer, this would seem to contradict the extensive structural evidence showing that the size of mature fragments is determined by the molecular distance between the PAZ and RNase III domains [69-72]. Imprecision in Drosha processing has been speculated to result in shifted-isomiRs [73], and while studies so far support a fixed-length cleavage of two helical turns [20], the experiments described here do not exclude imprecision as a contributing mechanism for isomiR biogenesis.

The use of the word 'imprecision' to describe isomiR production connotes images of waste, unnecessary by-products, and non-functional output - but if this were true, why would such wasteful 'imprecision' be maintained through millions of years of evolution? In the case of miR-10 and other human hairpins, more isomiRs than canonical miRNAs are produced. Data from this study and others argue against isomiRs as non-functional by-products - they are associated with both AGO complexes [45,54,55] and actively translating mRNAs (Figure 5). So what then is the biological function of these highly expressed isomiRs? The overlapping targeting (Figures 6c and 7) and high correlation of isomiRs (Figure S9 in Additional file 1) with their canonical miRNAs may answer one of the most intriguing paradoxes of miRNA function: the ability to drive specific biological phenotypes given the flexible nature of miRNA-mRNA interactions. As few as six nucleotides are required in the 'seed' region to induce repression (and/or degradation), and mismatches, bulges, and G:U wobble can all be tolerated if there is sufficient compensatory binding at the 3' end. Additionally, if there is a sequence match to the centered site, homology at the 5' end of the miRNA is not required for function. The combination of these varied potential interactions leads directly to the prediction that miRNAs could target hundreds, if not thousands, of mRNAs, a prediction that has been confirmed by biotin pull-down assays in this study. Intriguingly, despite this apparent lack of specificity, miRNAs are involved in specific biological roles. Clues to how this specificity is achieved have been seen previously, with miRNAs targeting functionally related genetic networks either alone [64] or co-operatively with other miRNAs [74]. However, these genetic networks are small when compared to the hundreds of genuine mRNA targets of any given miRNA. Simply increasing the dosage of a single miRNA would not be expected to improve the 'on-target' to 'off-target' ratio; however, the addition of similar molecules with different repertoires of mRNA targets would distribute the 'off-target' hits while still targeting the core biological networks (Figure 8). Indeed, we did find a statistically significant enrichment for multiple miRNA targeting of the core miR-10 network; however, it is important to remember that we have profiled only one isomiR from each hairpin, where around 20 have been detected above the expression threshold. Not only would the isomiR-driven signal to noise ratio improve the sensitivity of the miRNA-controlled networks, but it could also increase exponentially the dynamic range of such targeting as combinatorial suppressive effects are exploited. An important caveat is that the hairpins studied here have more than 50% of their output as isomiRs. Such a model will probably not be applicable to hairpins where the proportion of isomiRs is low. However, there is scope for this model to be more widely applicable - when considering individual isomiRs, approximately 23% have expression the same as or greater than that of the canonical miRNA (Figure 3c). Further study would be required to explore the extent that this model could contribute to gene regulation.

While the results shown here have emphasized what is similar between isomiR and canonical miRNA targeting, it is also possible that the differences could be biologically meaningful. An alternative (but not mutually exclusive) hypothesis is that isomiRs fine tune mRNA targeting to the requirements or responses of an individual cell type, and that cell-type-specific isomiR profiles may be invisible in our profiling of whole organs. This is not an easy hypothesis to test in the whole-organ profiling we have performed for this study, and is likely to require in-depth sequencing on individual cell types. At this stage, we are also unable to answer questions regarding the specific binding sites through which isomiRs target their mRNAs, and whether or not they competitively or cooperatively regulate their target mRNAs. Substantially more information about the wiring of these genetic networks could be gained by understanding exactly which miRNA binding sites are being occupied, something not currently possible with the microarray approach described here. Such attempts would be confounded by both the necessity to computationally infer potential binding sites, and the reliance on a reference genome to predict the presence of a binding site in the transcriptome of a cancer (and possibly highly mutated) cell line. Future studies should include simultaneous profiling of mRNA and miRNA populations across a number of tissues to determine the co-expression of miRNAs and their predicted targets, while systematic and comprehensive studies into individual isomiR targets and their precisely localized and experimentally confirmed binding sites will undoubtedly shed light on the hypotheses presented here.

## Conclusions

IsomiRs have been reported in almost every sequencing-based study published to date, and yet their functional significance remains controversial. Our results demonstrate that isomiRs are functional, and that their biological role is likely to involve improving the signal to noise ratio in miRNA-mRNA targeting. These results have substantial implications for understanding the precise molecular mechanisms behind miRNA-controlled genetic networks, as the best target prediction programs are sensitive to a precise definition of a seed sequence, which can vary in isomiRs. However, incorporating knowledge of isomiR targets into systems biology approaches will greatly increase the sensitivity of miRNA-mRNA network discovery.

## Materials and methods

### Library preparation and sequencing

Construction of small RNA libraries was performed using both the Small RNA Expression Kit (SREK, Ambion, Austin, TX, USA), and the Whole Transcriptome Amplification Kit (WTAK, Ambion) according to the manufacturer's instructions. Briefly, a mixture of both 5' and 3' adaptors was combined with flashPAGE™ purified small RNAs (approximately 15 to 40 nucleotides) from FirstChoice^® ^total RNAs (Ambion), and simultaneously ligated in a single reaction. cDNA was synthesized, and PCR amplified. During the PCR, sequencing adaptors were incorporated along with a unique six-nucleotide barcode sequence. Following library construction and purification, barcoded libraries were pooled and sequenced together using the Applied Biosystems (Beverly, MA, USA) SOLiD system.

These two protocols both use random hexamers attached to double-stranded adaptors to capture and position the small RNA for efficient ligation. SREK uses the 3' hexamer adaptor to prime cDNA synthesis, whereas WTAK uses a separate primer, and results in a different profile or errors in the 3' end of the miRNA. By examining the substitution isomiRs that are present in both libraries of the same tissue, we can exclude those that may be artifacts of library construction [SRA:SRP006043].

### Quantitative reverse-transcriptase PCR

Triplicate reverse transcription reactions were done on total RNA samples using both the A and B Megaplex™ RT primer pools in conjunction with the TaqMan^® ^MicroRNA Reverse Transcription Kit following the manufacturer's protocol. Reactions were run on an Applied Biosystems 7900HT. All arrays were analyzed using manual C_T _with automatic baseline adjustment and the threshold set at 0.1. A miRNA was considered to be detected by TaqMan if the average C_T _was < 35.

### miRNA and isomiR analysis with miRNA-MATE

Small RNA sequencing tags were aligned using miRNA-MATE v1.1, an open source alignment tool designed in our laboratory specifically for color-space miRNA analysis. The pipeline is written in Perl, and source code, test data, and a comprehensive user manual are available to download from [75]. miRNA-MATE uses the recursive style of matching [50] for sensitive miRNA expression detection, but also can identify and strip the adaptor sequence to determine the precise ends of the captured miRNAs. Given the error rate of massive-scale sequencing (even when using the error-correcting properties of 2-base encoding by SOLiD) and the need to identify sequence-variant isomiRs, alignment against the entire genome would be impractical. Using vmatch [76], and allowing two nucleotide substitutions (no insertions or deletions), only 335 (30.5%) human miRNAs could be uniquely placed against Hg19 (Table S2 in Additional file 1). Instead, we have restricted the search space to include only annotated pre-miRNA hairpin sequences (miRBase v15), and have allowed for up to two nucleotide substitutions during the alignment. The minimum length of tags matched was 20 nucleotides, as the specificity of matching drops dramatically at shorter lengths (data not shown). Tags were also matched using the same parameters to the complete repertoire of mature miRNA sequences from all species (miRBase v15) to determine the false positive matching rate for unrelated species. Sequences of rRNAs, tRNAs, and snoRNAs were downloaded from the UCSC Genome Browser [77], and aligned to as described above for miRNAs.

Wild-type and catalytically inactive AGO2 mutant miRNA-seq profiles [33] were downloaded from the NCBI Short Read Archive (accession number [SRP002411]). Fastq files were converted to color-space using a custom perl script, and mapped to *D. rerio *pre-miRNA hairpins (miRBase V17) using miRNA-MATE as described above. The two wild-type libraries (SRR042431 and SRR042433) were combined, but the depth of sequencing was still approximately ten times shallower than the mutant libraries. To correct for this massive sampling bias, we resampled the mutant library to produce a data set of the same depth as the wild-type library.

To correct for the distortion of proportional measures of expression (such as transcripts per million) that can happen when comparing very different expression profiles or libraries, we applied the trimmed mean of M-values scaling method [78]. All transcript per million (tpm) values reported in this manuscript have been corrected for these effective library sizes.

### Total, RNA-associated and polysome-associated miRNA preparation

To prepare polysome-associated miRNAs, approximately 6 × 10^7 ^HeLa cells were incubated with 10 μg/ml cycloheximide for 10 minutes at 37°C, prior to lysis in hypotonic lysis buffer (10 mM KCl, 1.5 mM MgCl_2_, 10 mM Tris-Cl, pH 7.4, 10 μg/ml cycloheximide) and allowed to swell on ice for 10 minutes. Cells were homogenized using a 21 gauge needle, and centrifuged at 2,000 g for 2 minutes at 4°C to pellet nuclei and unlysed cells. The lysate was brought to a total volume of 400 μl with gradient buffer (150 mM KCl, 5 mM MgCl_2_, 50 mM Tris-Cl, pH 7.4). We prepared two gradients in Ultra-Clear Thinwall 2.2 ml 11 × 34 mm centrifuge tubes (Beckman Instruments Inc., Fullerton, CA, USA), consisting of 1 ml of 1.5 M sucrose in gradient buffer, then 1 ml of 0.5 M sucrose in gradient buffer, and finally 200 μl of cell lysate. The gradients were spun at ≥ 250,000 g for 5 hours at 4°C. Fractions (60 μl) were removed from the top of the gradient, and the UV absorbance was measured at 260 nm. Fractions containing polysomes were pooled, and RNA was extracted using an RNeasy kit (Qiagen, Doncaster, VIC, Australia) according to the manufacturer's instructions. miRNAs were eluted from the mRNAs by heat (95°C for 2 minutes), and purified from the large RNAs using flashPAGE Fractionator and the flashPAGE Reaction Clean-up Kit (Ambion) according to the manufacturer's instructions.

RNA-associated miRNAs were collected by purifying total RNA from HeLa cells using an RNeasy column (Qiagen) according to the manufacturer's instructions. miRNAs were eluted as for polysome-associated miRNAs. Total HeLa miRNAs were purified using a miRNeasy column (Qiagen) according to the manufacturer's instructions. All miRNAs were then captured and sequenced as described above.

To confirm that miRNAs were not retained on the RNeasy columns in the absence of long RNAs, total RNA processed with both miRNeasy and RNeasy columns were heat denatured for 2 minutes at 75°C, and re-purified through a new miRNeasy or RNeasy column, respectively. RNeasy purified RNA was also re-purified through a new RNeasy column without heat denaturation. cDNA was prepared using a TaqMan MicroRNA Reverse Transcription Kit (Life Technologies, Foster City, CA, USA). qRT-PCR was performed using SYBR green PCR master-mix (Life Technologies) on an ABI 7900HT 7 Real time PCR system. Expression values were normalized to RNU6B.

### Biotinylated miRNA pull-downs

Synthetic biotin-labeled miRNA duplexes (200 pmoles; Table S8 in Additional file 1; Integrated DNA Technologies San Diego, CA, USA) were transfected into 4 × 10^6 ^HEK293T cells using HiPerFect Transfection Reagent (Qiagen). Cells were harvested after 24 hours, and lysed in hypotonic lysis buffer (10 mM KCl, 1.5 mM MgCl_2_, 10 mM Tris-Cl pH 7.5, 5 mM DTT, 0.5% NP-40, 60 U/ML SUPERase•In (Ambion) and 1× Complete Mini protease inhibitor (Roche, Dee Why, NSW, Australia). Cell debris was cleared by centrifugation (≥ 10,000 g at 4°C for 2 minutes). The supernatant was transferred to a clean tube, and NaCl was added to a final concentration of 1 M. myOne C1 Dynabeads (25 μl; Invitrogen, Thornton, NSW, Australia) were pre-blocked with 1 μg/μl bovine serum albumin and 1 μg/μl yeast tRNA (Invitrogen), and incubated with the supernatant for 30 minutes at room temperature. Beads were then washed with hypotonic lysis buffer and 1 M NaCl before RNA extraction using an RNeasy Kit (Qiagen) according to the manufacturer's instructions.

### Microarray hybridizations and analysis

MicroRNA microarray samples were prepared using the Agilent miRNA Microarray System protocol. An input of 100 ng total RNA was labeled and hybridized to the Agilent Human miRNA array (AMADID 016436) for 20 hours at 55°C. The slides were scanned using the Agilent Scanner G2505B US22502591 (Agilent Technologies) and data were extracted using the Feature Extraction software v9.5.3.1 (Agilent Technologies).

Fifty nanograms of the mRNA samples captured in the biotin pulldowns were amplified and labeled using the Illumina TotalPrep RNA Amplification Kit as per the manufacturer's instructions. Post-amplification RNA quality control was performed using a NanoDrop (Thermo Scientific, Bellefonte, PA, USA) and an Agilent 2100 Bioanalyzer. Amplified RNA (750 ng) was hybridized onto Illumina human HT-12 chips (V3 and V4), and scanned on a Bead Array Reader (Illumina) [GEO:GSE29101]. Expression measurements were extracted using the GenomeStudio (version 2009.1; Illumina) software. The data from all microarrays were first normalized using the median percentile rank approach [77]. Thresholds of 0.76 and 0.95 were selected for miR-17-5p and the miR-10 family, respectively, and genes that had that rank or higher were selected for GSEA. GSEA was performed using Ingenuity Pathway Analysis [79].

### Accession numbers

The sequencing data reported in this study can be obtained from the NCBI Short Read Archive (SRA) under accession number [SRP006043], and the microarray data are available from the Gene Expression Omnibus (GEO) under accession number [GSE29101].

## Abbreviations

AGO2: argonaut 2; ac-pre-miRNA, argonaut cleaved precursor miRNA hairpin; GSEA: gene set enrichment analysis; miRNA: microRNA; miRNA-seq: microRNA sequencing; NCBI: National Center for Biotechnology Information; pre-miRNA: precursor microRNA hairpin; qRT-PCR: quantitative reverse-transcriptase polymerase chain reaction; RISC: RNA induced silencing complex; RNA-seq: RNA sequencing; snoRNA: small nucleolar RNA; SREK: Small RNA Expression Kit; tpm: transcripts per million; WTAK: Whole Transcriptome Amplification Kit.

## Competing interests

JG, KL, SH, CB, XW, CL, JI, KM, KB, and SK are currently or were previously employed by Life Technologies, a manufacturer of sequencing instrumentation and reagents. No other authors have any competing interests.

## Authors' contributions

NC conceived and designed the experiments, performed the experiments, analyzed the data and wrote the manuscript. SK and SMG conceived and designed the experiments and wrote the manuscript. SW, JG, KL, SH, ALS, EN, KK, BBG, and KN performed the experiments. QX, CB, HCM, XW, JAS, NM, DLW, KSK, NW, and JS analyzed the data. CL, JI, KM, and KB contributed reagents, materials and/or analysis tools. All authors have read and approved the manuscript for publication.

## Supplementary Material

Additional file 1 **Supplementary figures, figure legends and tables**. Click here for file

Additional file 2 **miRNA-MATE source code and user manual**. Click here for file

Additional file 3 **A description of the proposed nomenclature for isomiRs**. Click here for file

Additional file 4 **Tab-delimited text file of the raw counts of isomers detected in the libraries created in this study**. Click here for file
