# Supplementary material for: MicroRNAs and their isomiRs function cooperatively to target common biological pathways
Source: Genome Biol. 2011 Dec 30;12(12):R126. doi: 10.1186/gb-2011-12-12-r126 (PMC3334621; doi:10.1186/gb-2011-12-12-r126)
Supplement: Additional file 1 — Supplementary figures, figure legends and tables. [file gb-2011-12-12-r126-S1.PDF]

Supplementary Figures and Tables

This document contains all supplementary figures and table associated with the manuscript “MicroRNAs and their isomiRs function cooperatively to target common biological pathways.”.

Supplementary Figure Legends

Supplementary Figure S1. Owing to their small size, mature miRNAs contain limited information for mapping. (A) The size distribution of known miRNAs from miRBase 15.0 showing that most known miRNAs are 21-23nt long. (B) Mapping specificity is systematically lower for shorter tags. Every possible (non-‘N’ containing) genomic sequence of a defined length (on the x-axis) was taken directly from chromosome 13 at 1nt intervals, and mapped back to the complete human genome (hg19). Grey bars represent the proportion of uniquely mapping tags when no mismatches were allowed. Black bars represent the proportion of uniquely mapping tags when 1 colour-space mismatch was allowed, highlighting a systematic decrease in the shorter tags. This problem is amplified as the number of allowed mismatches increase.

Supplementary Figure S2. The miRNA-MATE pipeline. The pipeline uses two alignment strategies: recursive (1-3) and adapter trimming (1, 4-8). (1) Optionally, samples are identified by a unique barcode sequence and split accordingly – this step is

not required in the later . (2) Tag is aligned to a custom reference library (miRBase v15 pre-miRNA hairpins for this study), if tag doesn’t align iterative truncation is performed until tag aligns, or the minimum length (20nt for this study, but this can be configured) is reached. (3) miRNAs are counted and tabulated. (4) The adaptor sequence is identified in each tag, and the adaptor and the colour-space transition base are chopped. (5) Tags are aligned to a custom reference library at their exact size only. If they do not align at this length, then the tags are discarded. (6) Aligned tags are converted from colour-space to base space using the reference sequence. (7) IsomiR specific information is summarized in tabular format. (8) Sequence logos from each reference segment (pre-miRNA hairpins for this study) are created.

Supplementary Figure S3. Determining the threshold for noise in our miRNA-seq data. Aligning tags to a reference library containing mature miRNAs from all available species showed that a baseline of 10 tpm is sufficient to exclude the vast majority of sequencing error, whilst being sensitive enough to detect isomiR expression. Means and standard deviations of miRNA expression in each species are plotted.

Supplementary Figure S4. Hierarchical clustering of miRNA gene expression profiles. The 470 mature miRNAs expressed at > 10 tpm in at least one tissue were included. Median centered relative expression for each miRNA was subjected to hierarchical clustering by Pearson correlation using the average linkage clustering algorithm. These results show that the samples cluster by tissue of origin, confirming that the threshold

was set at a level appropriate for the dataset. Full details of RNA batches, library preparation methods, and sequencing runs can be found in Supplementary Table 1.

Supplementary Figure S5. Correlation between miRNA-Seq and TaqMan LDA data for 9 tissues using the recursive matching strategy. In all plots, points represent the mean, and bi-directional error bars represent standard error. ρ values indicated are Spearman correlations.

Supplementary Figure S6. Correlation between miRNA-Seq and TaqMan LDA data for 9 tissues using the adaptor trimming strategy to identify canonical sequences. In all plots, points represent the mean, and bi-directional error bars represent standard error. ρ values indicated are Spearman correlations.

Supplementary Figure S7. Global analysis of insertion and deletions events. (A) Distribution of lengths where deletions occur within a tag relative to the reference sequence. (B) Distribution of lengths where insertions occur within a tag relative to the reference sequence. The breakdown of indel events for each tissue is indicated by the legend.

Supplementary Figure S8. Quality control of polysome fractionation RNA samples. (A) Spectral plots of RNA concentrations of duplicate polysome fractionation experiments. Each fraction of the two gradients was measured by spectrophotometer (A260nm). The fractions corresponding to polysomes is indicated by the red bar. (B) Bioanalyser profiles of the three RNA samples used to prepare miRNA-seq libraries. RINs (RNA Integrity

Numbers) of greater than 9.0 represent intact RNA. RNA from the polysome duplicates above was pooled to create a single RNA sample. (C) Top: Experimental design to confirm that miRNAs can stably associate with longer mRNAs, and are not retained on RNeasy columns in the absence of longer RNAs. Numbers in black circles represent the designated sample number used in qRT-PCR. Bottom: Expression of miRNAs detected by qRT-PCR relative to the expression level found in sample 1. Bars represent the mean of three technical replicates, and error bars are sem. The absence of miRNA expression in Sample 2 confirms that miRNAs are specifically associated with longer RNAs, and not the RNeasy column. (D) Bar graph depicting the relative expression of various RNA classes in the three HeLa samples used in this study.

Supplementary Figure S9. Correlation of isomiR expression with expression levels of corresponding canonical miRNAs. Bar and whisker plots of isomiR Pearson correlations show high concordance between isomiR and canonical miRNA expression. The right panel is a magnification of the left panel between 0.8 and 1.0.

Supplementary Figure S10. The biotin pull-down experiments enrich for previously validated targets. (A) The solid black line represents the frequency of genes with a defined median percentile rank" (x-axis). Mixture modeling was performed in MatLab to derive two normally distributed components (red and green lines) that fit the observed data (dashed black line). A threshold of 0.76 was selected to provide a cutoff with ~5% false discovery rate (thin dotted line). The ranks of 34 mRNA targets previously validated by luciferase assays are shown in the blue triangles. (B) GSEA of mRNAs present in

miR-17-5p biotin pull-downs compared with predicted miR-17-5p targets (TargetScan) and random gene lists of equivalent size. The enrichment of “cell death”, “cell cycle”, and “cellular proliferation” ontologies confirmed the known biology for this miRNA in regulating cell cycle progression, illustrating the specificity of the miRNA pull-down assay.

Supplementary Figure S1

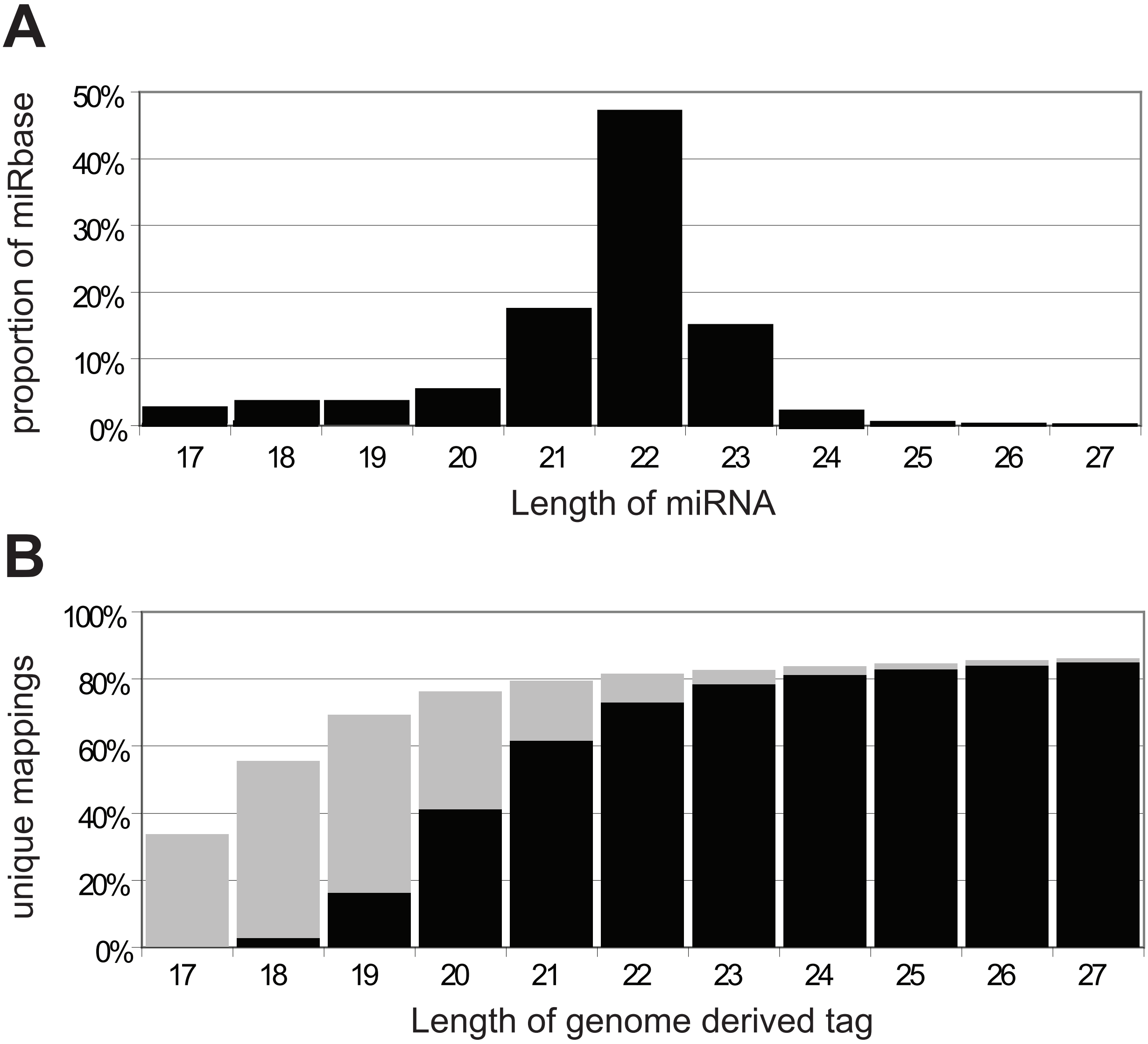

Supplementary Figure S2

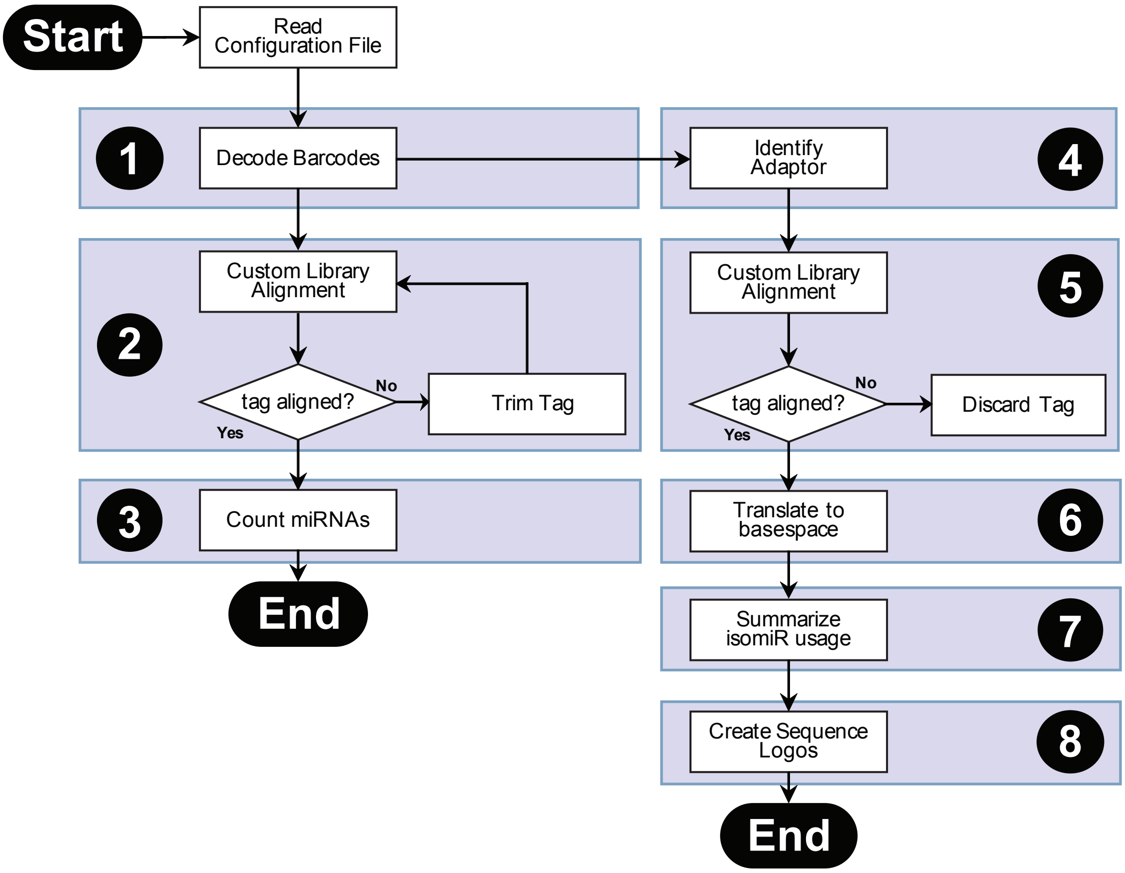

Supplementary Figure S3

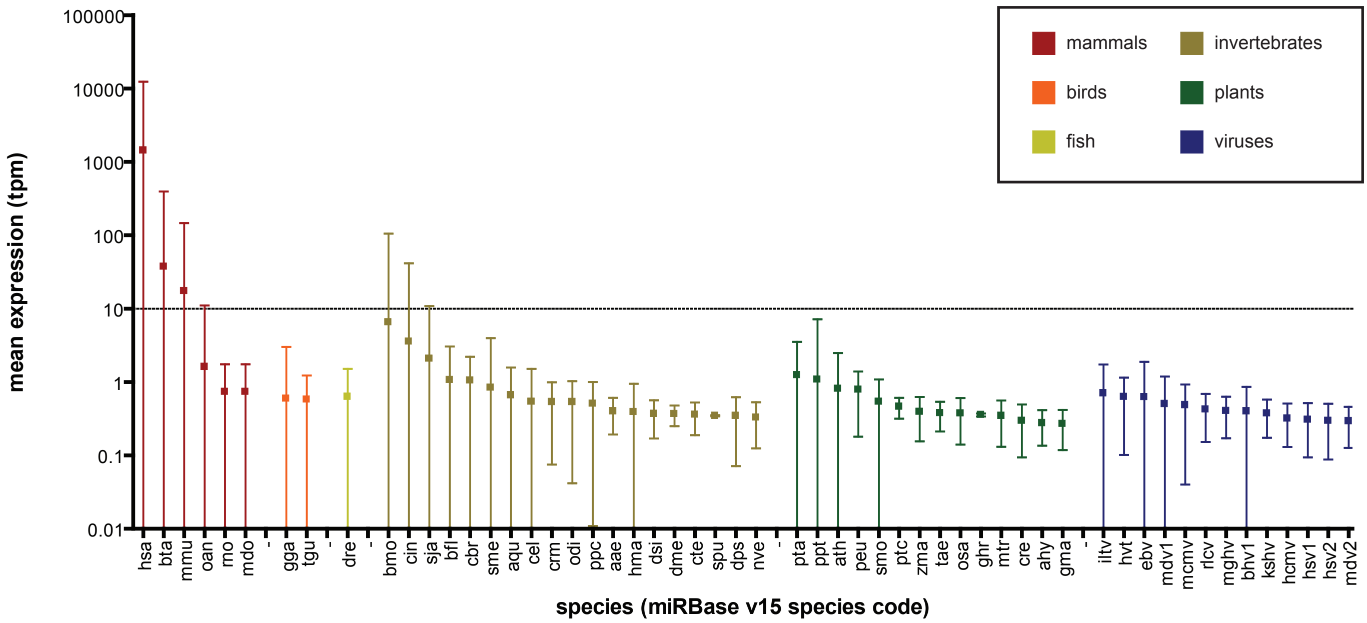

Supplementary Figure S4

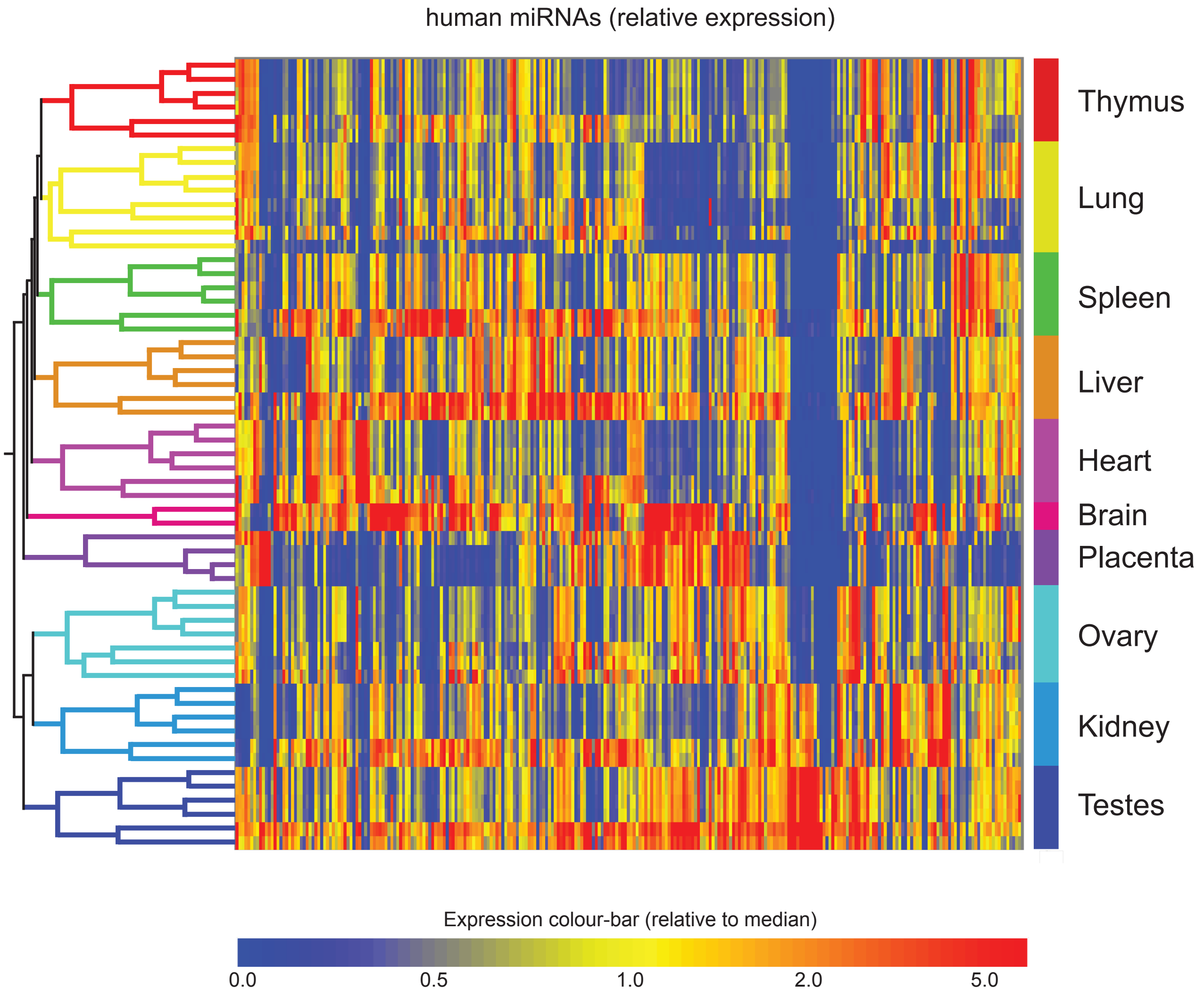

Supplementary Figure S5

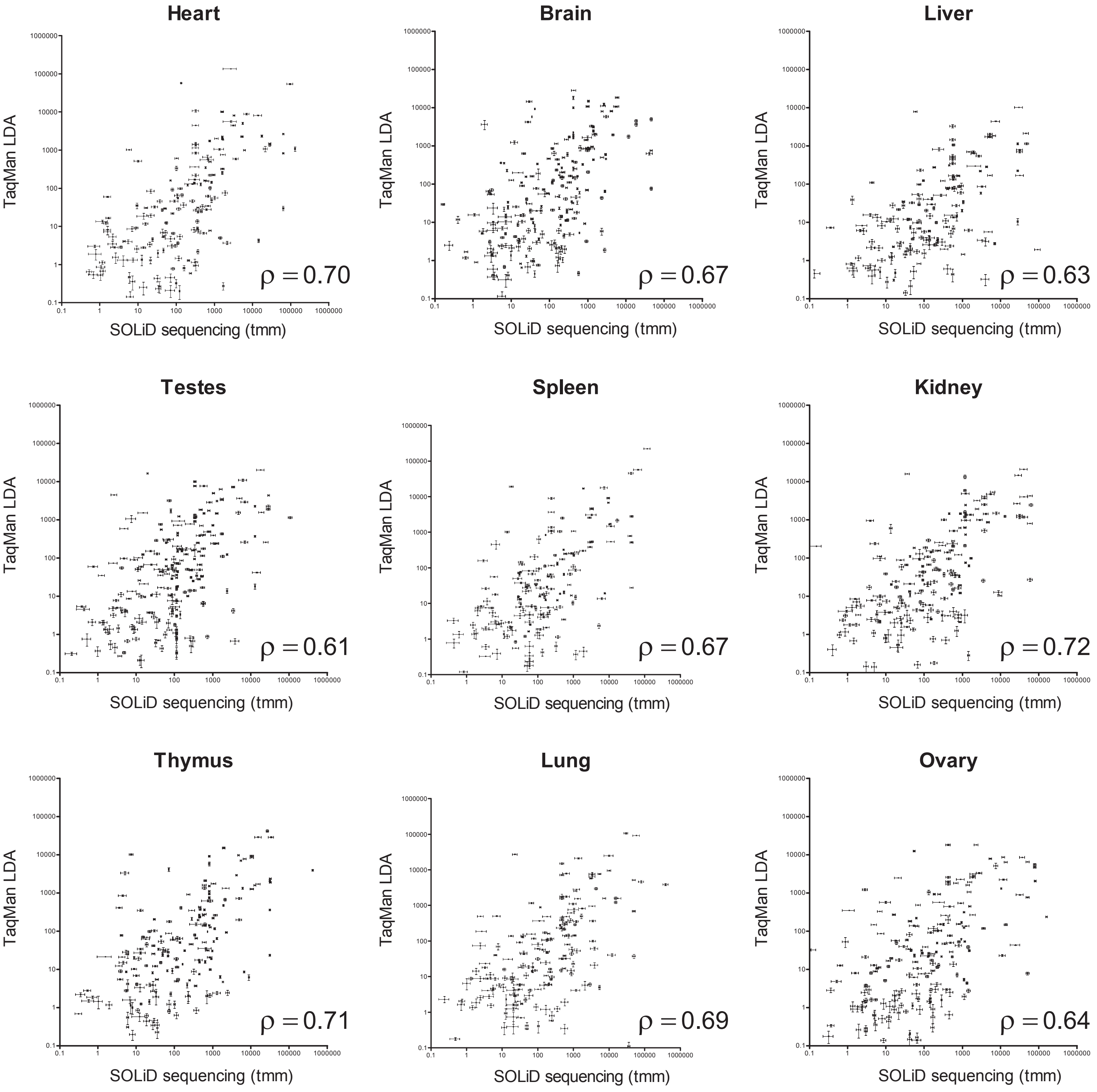

Supplementary Figure S6

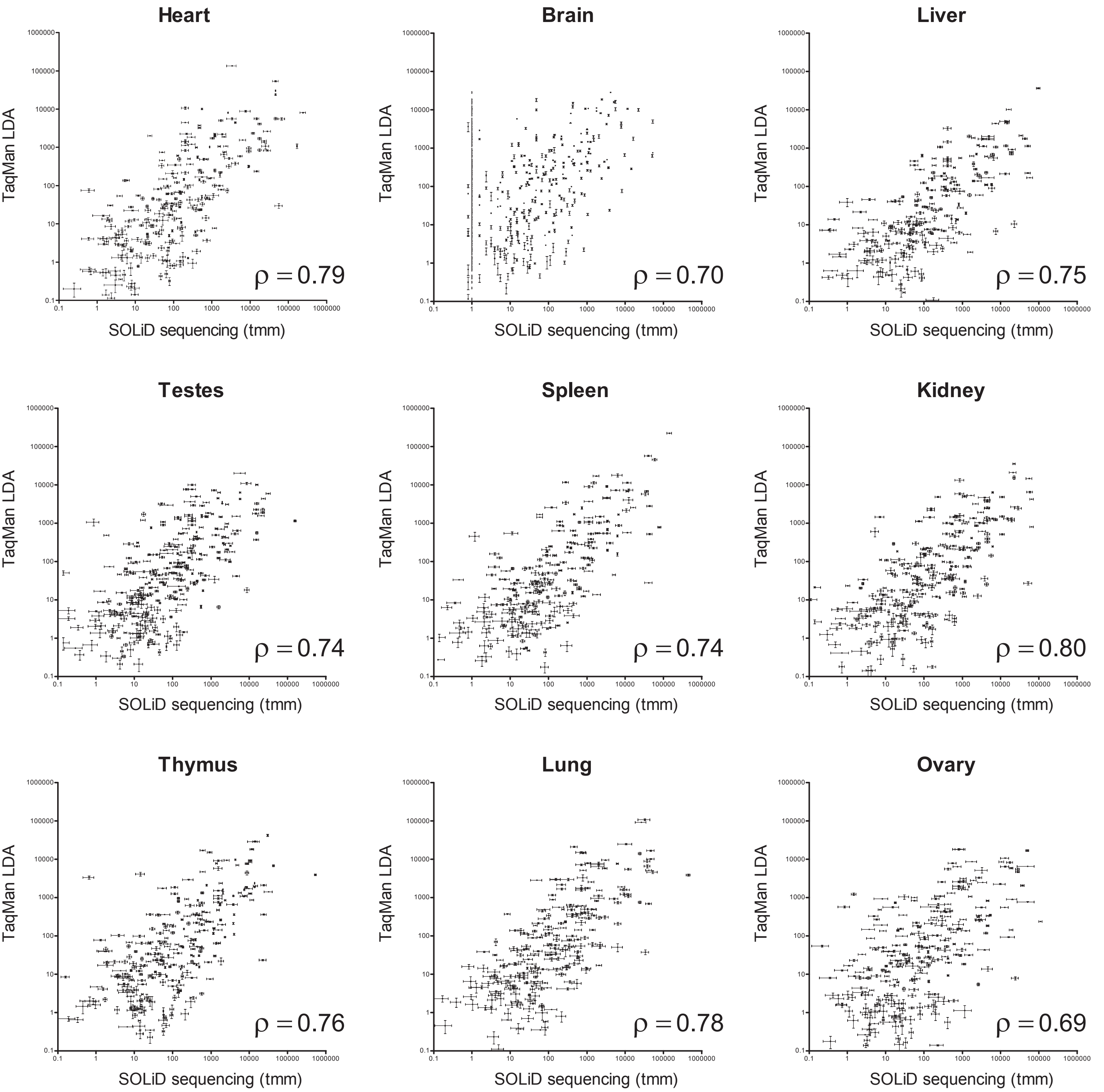

Supplementary Figure 7

A

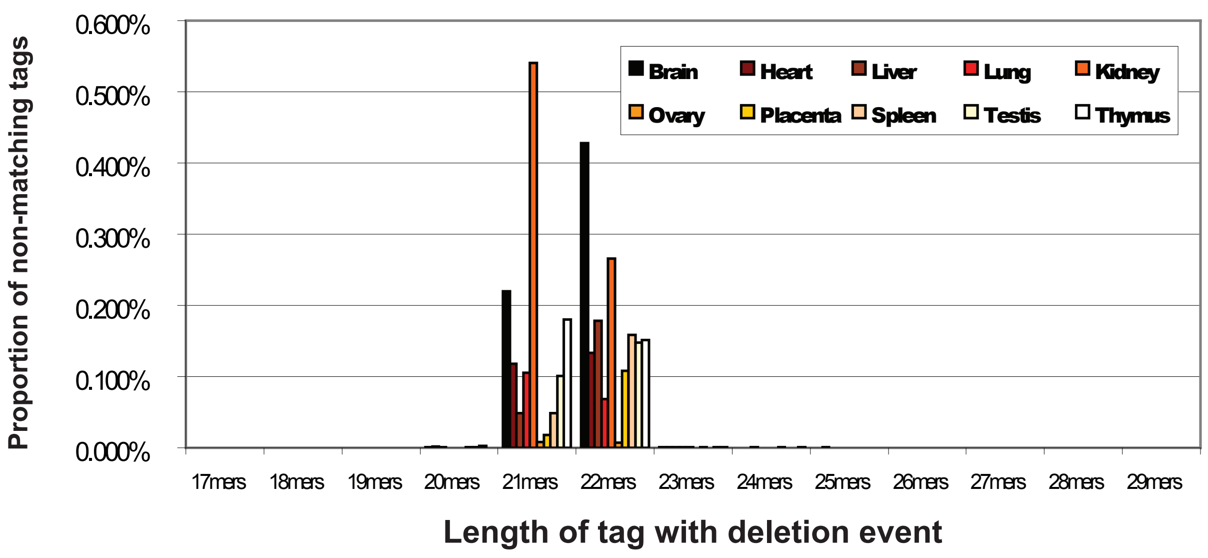

B

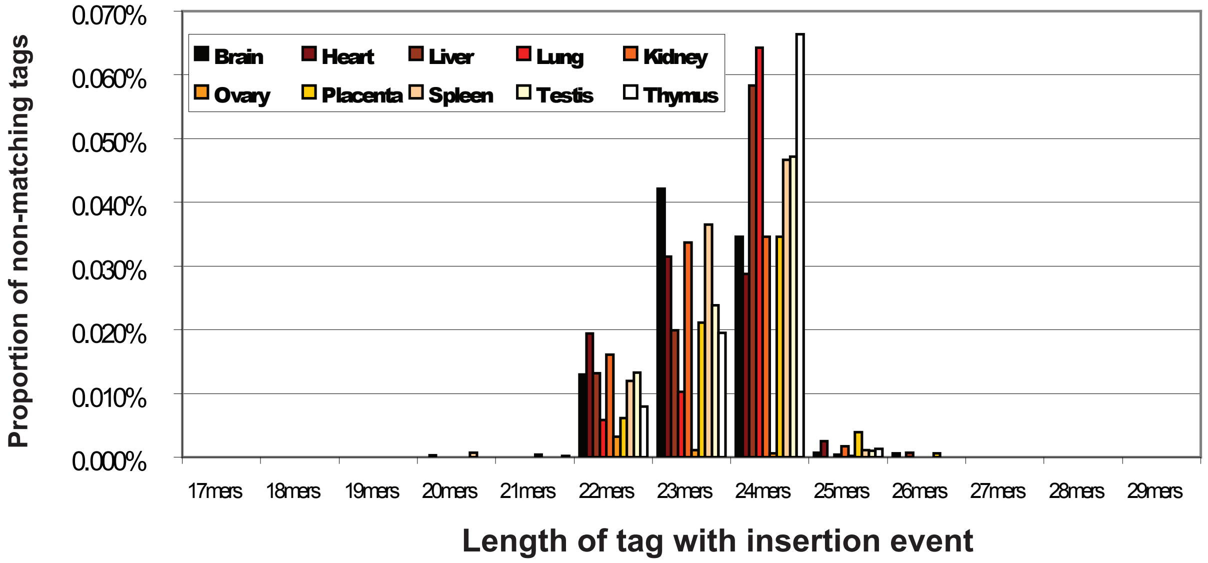

Supplementary Figure S8

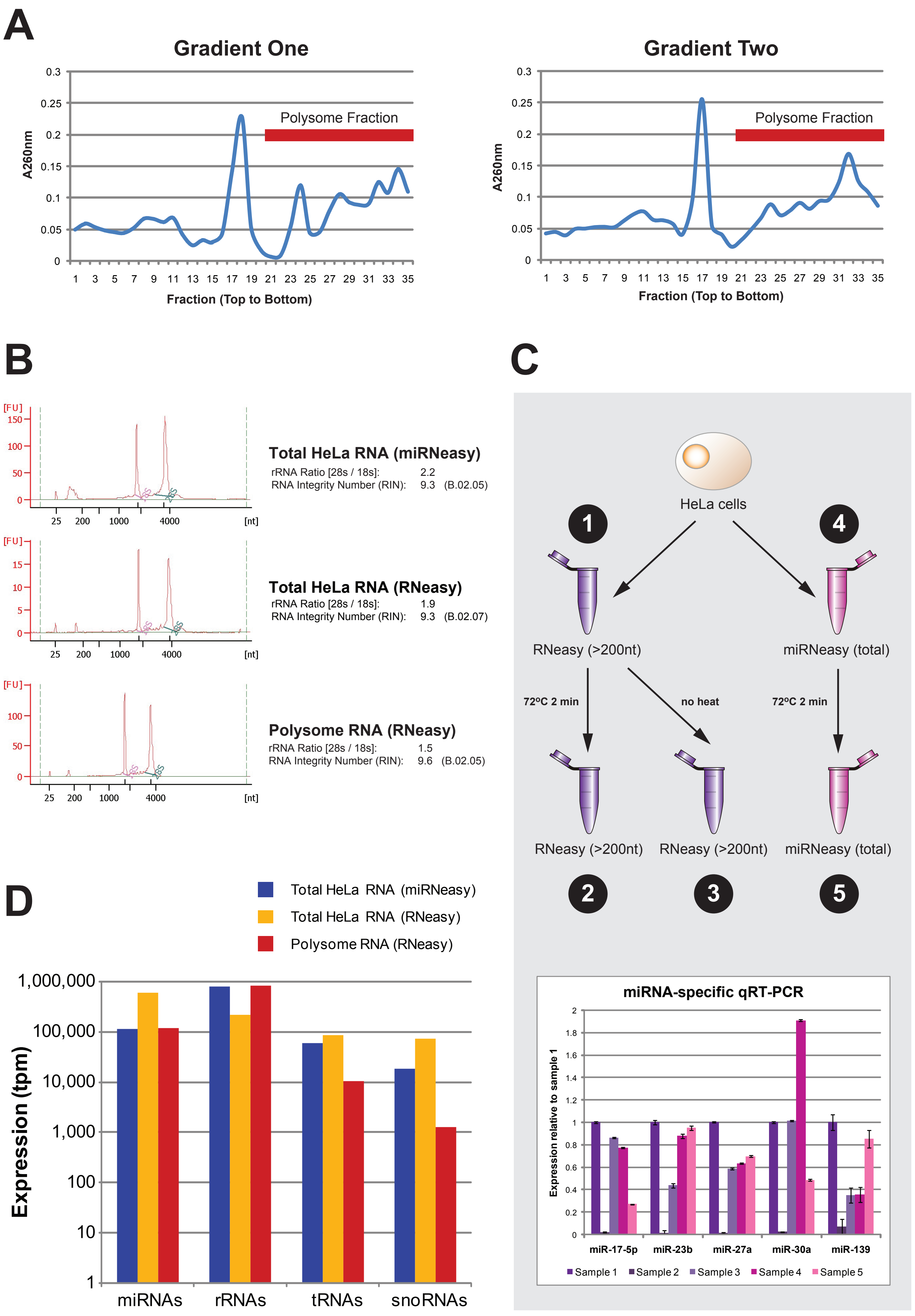

Supplementary Figure S9

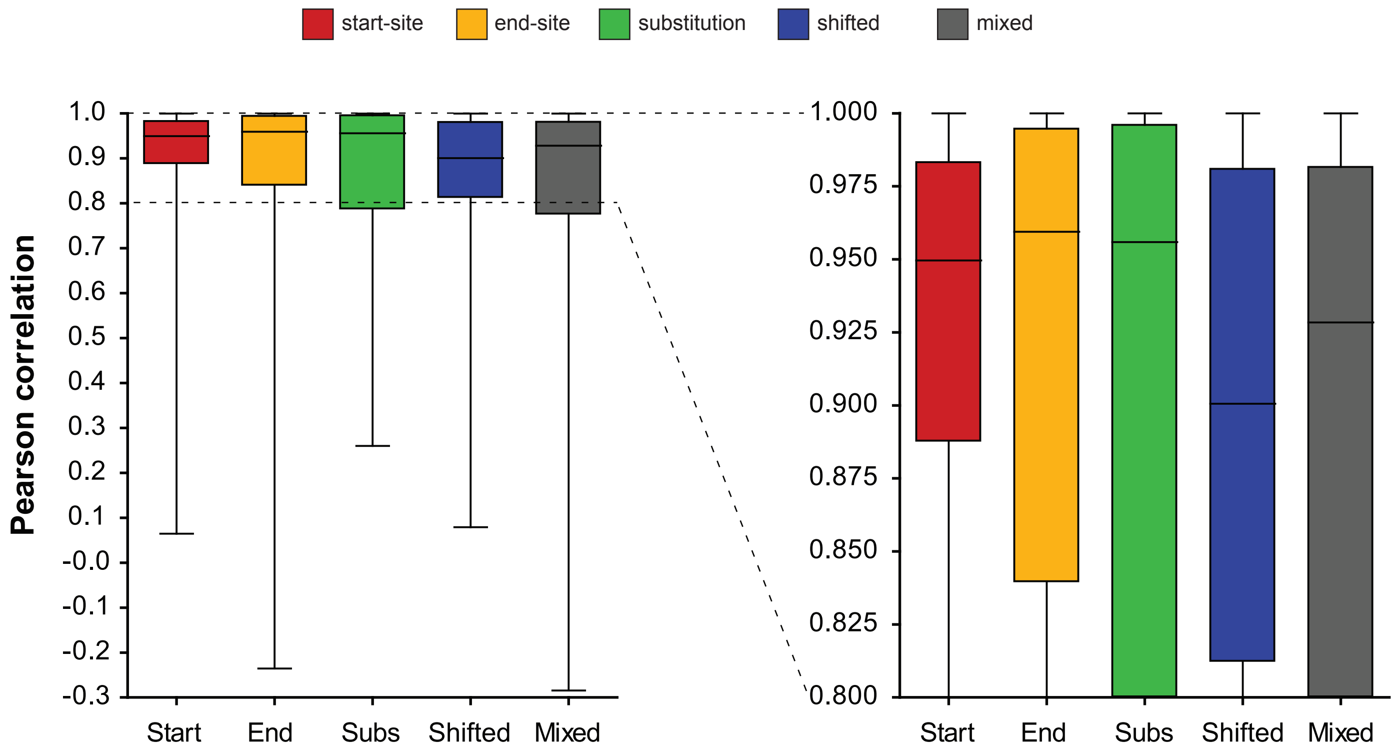

Supplementary Figure S10

A

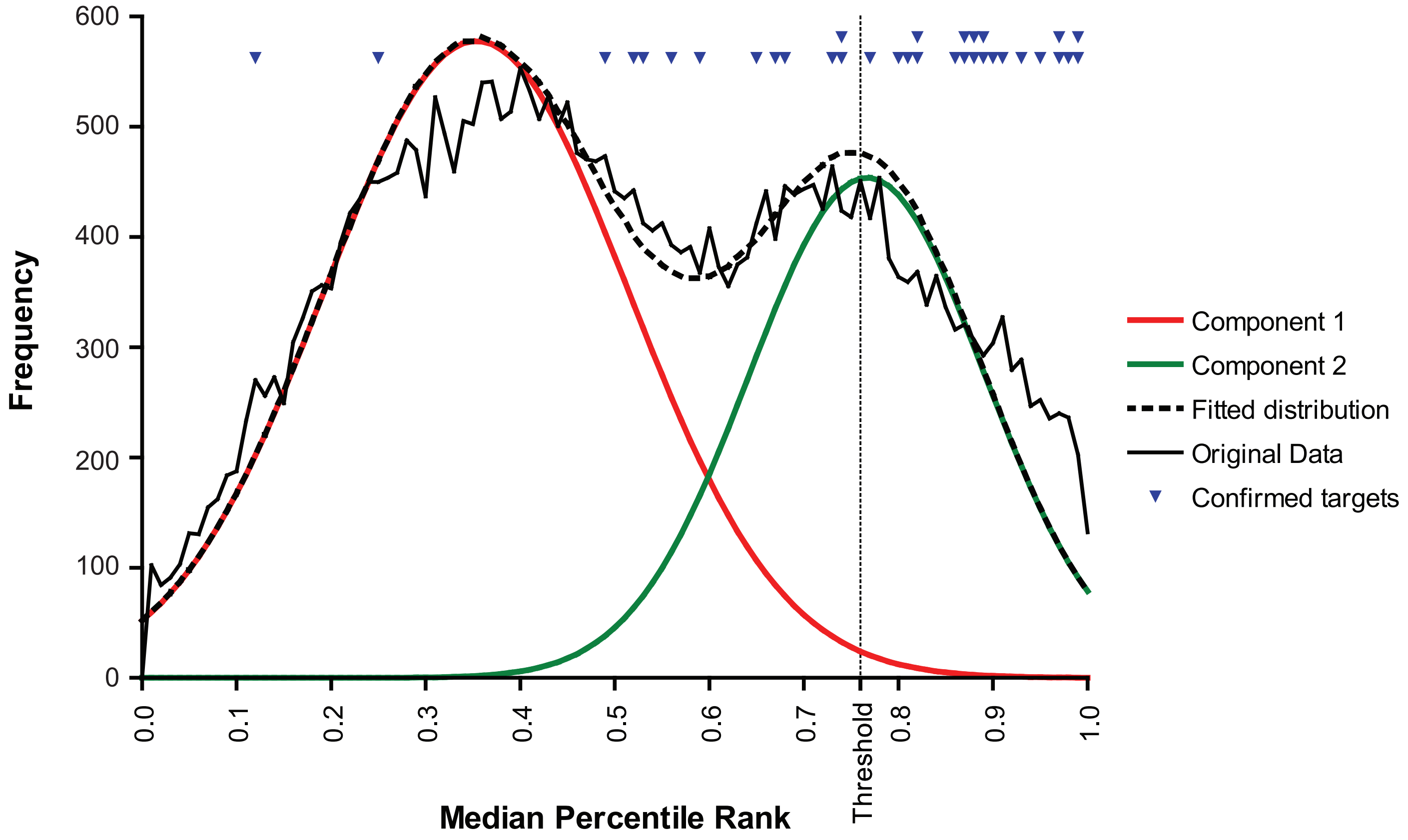

B

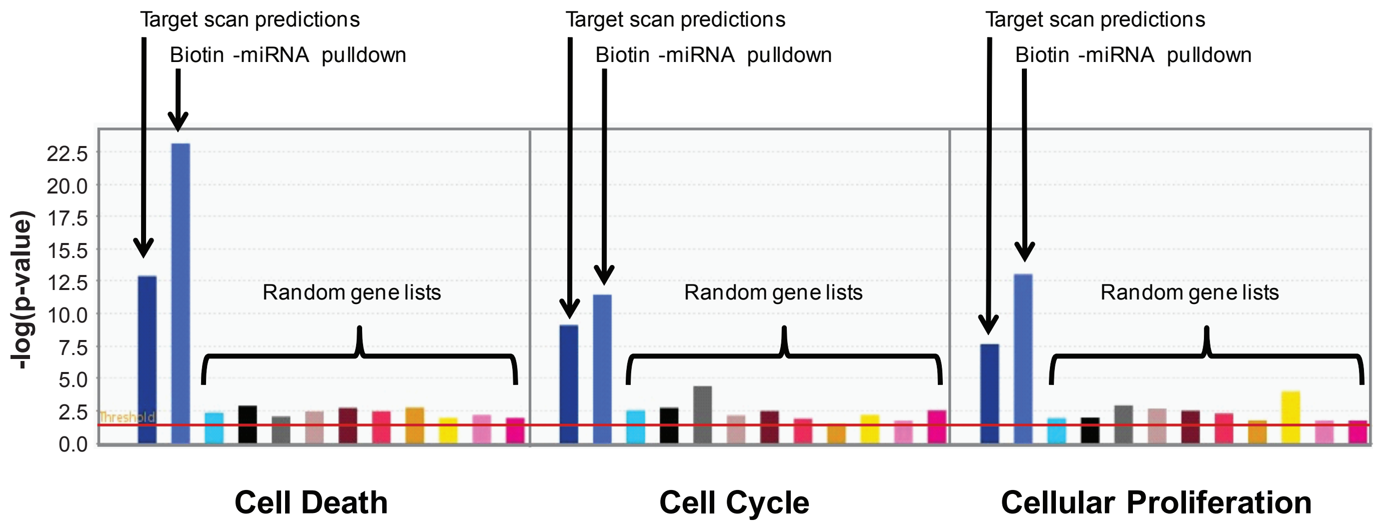

Supplementary Table S1. Sequencing statistics for small RNAs broken down to individual libraries.

Run Name	Prep	Library	Tissue	Donor ID (Lot #)	Mature mapped tags (recursive)	Mature mapped tags (adaptor trimming)	Hairpin mapped tags (recursive)	Hairpin mapped tags (adaptor trimming)
S0014_20080526_2_PolysomeAndAtlas_microRNA_Atlas	SREK	BC2	Brain	305098	1,606,496	1,241,131	2,295,549	1,321,577
S0014_20080902_1_uRNAandYogiFrag	SREK	BC2	Brain	305098	2,360,755	N/A	3,287,119	N/A
			**Brain Total**		**3,967,251**	**1,241,131**	**5,582,668**	**1,321,577**
B_20091223_SF35MP_BOURBON2_SREKVER_235TPA_FC1	WTAK	1_HT1	Heart	7020252	6,771,020	7,675,787	10,469,355	7,902,136
B_20091223_SF35MP_BOURBON2_SREKVER_235TPA_FC1	WTAK	2_HT2	Heart	7020252	5,584,047	6,328,215	8,727,061	6,317,474
B_20091223_SF35MP_BOURBON2_SREKVER_235TPB_FC2	WTAK	1_HT1	Heart	7020252	6,910,125	7,848,042	10,620,310	7,964,982
B_20091223_SF35MP_BOURBON2_SREKVER_235TPB_FC2	WTAK	2_HT2	Heart	7020252	5,546,364	6,231,924	8,509,087	6,172,689
S0014_20080526_2_PolysomeAndAtlas_microRNA_Atlas	SREK	BC1	Heart	7020252	1,278,700	1,222,099	2,581,389	1,327,171
S0014_20080902_1_uRNAandYogiFrag	SREK	BC1	Heart	7020252	1,999,768	N/A	3,831,207	N/A
			**Heart Total**		**28,090,024**	**29,306,067**	**44,738,409**	**29,684,452**
B_20091223_SF35MP_BOURBON2_SREKVER_235TPA_FC1	WTAK	7_KD1	Kidney	30300423	8,497,153	9,144,293	12,743,228	10,309,722
B_20091223_SF35MP_BOURBON2_SREKVER_235TPA_FC1	WTAK	8_KD2	Kidney	30300423	7,036,644	7,792,200	10,706,249	8,784,875
B_20091223_SF35MP_BOURBON2_SREKVER_235TPB_FC2	WTAK	7_KD1	Kidney	30300423	8,116,272	8,720,479	12,058,800	9,657,098
B_20091223_SF35MP_BOURBON2_SREKVER_235TPB_FC2	WTAK	8_KD2	Kidney	30300423	6,896,514	7,643,337	10,326,824	8,501,162
S0014_20080526_2_PolysomeAndAtlas_microRNA_Atlas	SREK	BC6	Kidney	70100119	1,155,520	825,909	2,086,826	1,303,970
S0014_20080902_1_uRNAandYogiFrag	SREK	BC6	Kidney	70100119	1,656,320	N/A	2,977,764	N/A
			**Kidney Total**		**33,358,423**	**34,126,218**	**50,899,691**	**38,556,827**
B_20091223_SF35MP_BOURBON2_SREKVER_235TPA_FC1	WTAK	3_LV1	Liver	10010031	5,065,390	5,356,347	7,881,997	6,273,264
B_20091223_SF35MP_BOURBON2_SREKVER_235TPA_FC1	WTAK	4_LV2	Liver	10010031	4,468,732	4,584,406	6,526,570	5,208,569
B_20091223_SF35MP_BOURBON2_SREKVER_235TPB_FC2	WTAK	3_LV1	Liver	10010031	5,102,041	5,461,957	7,800,266	6,257,891
B_20091223_SF35MP_BOURBON2_SREKVER_235TPB_FC2	WTAK	4_LV2	Liver	10010031	4,248,235	4,337,211	6,014,848	4,738,985
S0014_20080526_2_PolysomeAndAtlas_microRNA_Atlas	SREK	BC3	Liver	40000129	522,166	327,082	1,008,093	554,141
S0014_20080902_1_uRNAandYogiFrag	SREK	BC3	Liver	40000129	731,833	N/A	1,381,449	N/A
			**Liver Total**		**20,138,397**	**20,067,003**	**30,613,223**	**23,032,850**

B_20091223_SF35MP_BOURBON2_SREKVER_235TPA_FC1	WTAK	11_LU1	Lung	20100219	8,193,692	8,690,775	11,140,088	9,316,669
B_20091223_SF35MP_BOURBON2_SREKVER_235TPA_FC1	WTAK	12_LU2	Lung	20100219	7,504,187	8,726,138	11,783,057	9,514,038
B_20091223_SF35MP_BOURBON2_SREKVER_235TPB_FC2	WTAK	11_LU1	Lung	20100219	8,035,804	8,560,578	10,768,418	9,029,565
B_20091223_SF35MP_BOURBON2_SREKVER_235TPB_FC2	WTAK	12_LU2	Lung	20100219	7,978,629	9,127,246	11,995,629	9,764,576
LIZ_20080220_1	SREK	8087	Lung	20100219	2,470,695	1,663,346	3,877,219	2,099,206
R1a007_20080206_1_ABNmi_BC4a	SREK	BC4a	Lung	20100219	10,527,674	7,302,187	14,454,050	8,600,594
S0014_20080526_2_PolysomeAndAtlas_microRNA_Atlas	SREK	BC8	Lung	20100219	1,561,443	1,348,792	2,251,760	1,600,055
S0014_20080902_1_uRNAandYogiFrag	SREK	BC8	Lung	20100219	2,368,577	N/A	3,195,386	N/A
			**Lung Total**		**48,640,701**	**45,419,062**	**69,465,607**	**49,924,703**
B_20091223_SF35MP_BOURBON2_SREKVER_235TPA_FC1	WTAK	13_OV1	Ovary	808001	5,897,098	6,617,966	9,587,464	7,844,371
B_20091223_SF35MP_BOURBON2_SREKVER_235TPA_FC1	WTAK	14_OV2	Ovary	808001	5,135,174	5,857,029	8,645,952	7,021,514
B_20091223_SF35MP_BOURBON2_SREKVER_235TPB_FC2	WTAK	13_OV1	Ovary	808001	5,154,460	5,804,877	8,224,482	6,669,599
B_20091223_SF35MP_BOURBON2_SREKVER_235TPB_FC2	WTAK	14_OV2	Ovary	808001	5,087,948	5,806,480	8,405,422	6,780,646
Solid0039_20090211_2_Ovary_uRNA_and_ChIP_ovary_uRNA	SREK	BC1-2o	Ovary	808001	6,905,216	6,297,436	16,162,923	11,365,447
Solid0039_20090311_1_Ovary_ChIP_BC	SREK	BC1o	Ovary	808001	2,276,966	1,643,687	5,524,453	3,531,154
Solid0039_20090311_1_Ovary_ChIP_BC	SREK	BC2o	Ovary	808001	2,834,852	2,213,035	5,201,921	2,846,685
			**Ovary Total**		**33,291,714**	**34,240,510**	**61,752,617**	**46,059,416**
LIZ_20080220_1	SREK	8062	Placenta	02020094	1,366,635	1,067,856	2,440,187	1,285,241
R1a007_20080206_1_ABNmi_BC3a	SREK	BC3a	Placenta	02020094	6,973,743	5,311,276	8,954,384	5,035,967
R1a007_20080206_1_ABNmi_BC3b	SREK	BC3b	Placenta	02020094	7,970,198	6,467,232	10,442,436	6,105,886
R1a007_20080206_1_ABNmi_BC3n	SREK	BC3n	Placenta	02020094	8,247,283	6,735,728	10,760,789	6,338,158
			**Placenta Total**		**24,557,859**	**19,582,092**	**32,597,796**	**18,765,252**
B_20091223_SF35MP_BOURBON2_SREKVER_235TPA_FC1	WTAK	5_SP1	Spleen	30300423	6,601,309	7,244,963	9,513,115	7,713,302
B_20091223_SF35MP_BOURBON2_SREKVER_235TPA_FC1	WTAK	6_SP2	Spleen	30300423	6,799,681	7,565,629	10,105,612	8,066,101
B_20091223_SF35MP_BOURBON2_SREKVER_235TPB_FC2	WTAK	5_SP1	Spleen	30300423	6,685,429	7,300,291	9,418,619	7,696,861
B_20091223_SF35MP_BOURBON2_SREKVER_235TPB_FC2	WTAK	6_SP2	Spleen	30300423	6,918,014	7,680,934	10,102,189	8,107,714
S0014_20080526_2_PolysomeAndAtlas_microRNA_Atlas	SREK	BC5	Spleen	30300423	668,214	493,775	1,256,473	806,588
S0014_20080902_1_uRNAandYogiFrag	SREK	BC5	Spleen	30300423	943,374	N/A	1,748,289	N/A
			**Spleen Total**		**28,616,021**	**30,285,592**	**42,144,297**	**32,390,566**

B_20091223_SF35MP_BOURBON2_SREKVER_235TPA_FC1	WTAK	15_TE1	Testes	5060398	4,677,635	5,305,525	8,217,140	6,870,944
B_20091223_SF35MP_BOURBON2_SREKVER_235TPA_FC1	WTAK	16_TE2	Testes	5060398	5,312,926	5,685,352	8,260,839	7,082,049
B_20091223_SF35MP_BOURBON2_SREKVER_235TPB_FC2	WTAK	15_TE1	Testes	5060398	4,710,263	5,415,585	8,221,090	6,883,165
B_20091223_SF35MP_BOURBON2_SREKVER_235TPB_FC2	WTAK	16_TE2	Testes	5060398	5,211,665	5,575,061	7,972,085	6,767,055
S0014_20080526_2_PolysomeAndAtlas_microRNA_Atlas	SREK	BC4	Testes	5060396	647,764	477,092	1,245,391	782,091
S0014_20080902_1_uRNAandYogiFrag	SREK	BC4	Testes	5060396	977,814	N/A	1,799,810	N/A
			**Testes Total**		**21,538,067**	**22,458,615**	**35,716,355**	**28,385,304**
B_20091223_SF35MP_BOURBON2_SREKVER_235TPA_FC1	WTAK	10_TY2	Thymus	1070121	11,015,160	11,764,555	15,102,233	12,980,388
B_20091223_SF35MP_BOURBON2_SREKVER_235TPA_FC1	WTAK	9_TY1	Thymus	1070121	7,489,309	7,860,350	10,074,810	8,477,560
B_20091223_SF35MP_BOURBON2_SREKVER_235TPB_FC2	WTAK	10_TY2	Thymus	1070121	10,769,828	11,493,384	14,521,431	12,430,507
B_20091223_SF35MP_BOURBON2_SREKVER_235TPB_FC2	WTAK	9_TY1	Thymus	1070121	7,144,840	7,484,642	9,480,734	7,890,817
S0014_20080526_2_PolysomeAndAtlas_microRNA_Atlas	SREK	BC7	Thymus	1070124	1,462,148	1,280,694	2,199,498	1,499,244
S0014_20080902_1_uRNAandYogiFrag	SREK	BC7	Thymus	1070124	2,299,620	N/A	3,248,792	N/A
			**Thymus Total**		**40,180,905**	**39,883,625**	**54,627,498**	**43,278,516**

Supplementary Table S2. Number miRNAs from the small RNA tissue panel mapping uniquely to the human genome (hg19) using different alignment stringencies. Alignments were performed in base-space using vmatch.

	**0 mismatches**	**1 mismatch**	**2 mismatches**	**3 mismatches**
Uniquely-mapping	883	666	335	78
Multi-mapping	217	434	765	1022
**TOTAL**	**1100**	**1100**	**1100**	**1100**

Supplementary Table S3. The number of miRNAs or hairpins detected above the minimum threshold of 10 tpm. This analysis was performed by averaging all sequencing data from the same tissue together before calculating expression levels.

Tissue	Expressed mature miRNAs (recursive)	Expressed mature miRNAs (adaptor trimming)	Expressed hairpins (recursive)	Expressed hairpins (adaptor trimming)
Brain	259	291	325	273
Heart	251	283	308	264
Kidney	267	311	321	287
Liver	254	296	294	258
Lung	254	288	295	260
Ovary	254	297	305	268
Placenta	258	314	325	292
Spleen	255	290	288	255
Testes	277	322	334	289
Thymus	256	295	292	264
**TOTAL**	**408**	**470**	**470**	**423**

Supplementary Table S4. Table of human hairpins whose dominant arm changes over ten adult human tissues. Values represent the $\log_2$ of (average 5-prime expression)/(average 3-prime expression).

Hairpin	Brain	Heart	Kidney	Liver	Lung	Ovary	Placenta	Spleen	Testes	Thymus
hsa-miR-103-2	0.0	-1.1	-1.5	-2.8	-2.0	-0.4	1.0	-1.2	-1.1	-0.5
hsa-miR-126	-1.2	-0.7	-1.7	-0.8	-0.9	-0.9	2.0	-1.9	-0.3	-1.3
hsa-miR-136	1.9	1.6	2.1	2.2	1.2	1.3	-3.0	3.0	0.6	2.4
hsa-miR-154	-2.1	0.3	0.2	0.2	-0.6	2.1	-3.5	0.6	0.7	0.8
hsa-miR-190	-1.6	-0.6	-0.7	-1.2	1.1	0.5	1.6	0.9	0.5	-0.3
hsa-miR-1975	-1.3	-3.6	-1.6	-1.8	0.4	0.1	1.2	-2.0	-1.3	-1.3
hsa-miR-19b-1	-1.0	1.7	4.2	4.0	0.1	0.0	-2.4	4.0	2.4	2.9
hsa-miR-29b-2	-2.8	-0.5	1.2	0.0	-2.6	-1.7	-5.5	0.3	0.3	0.1
hsa-miR-3065	-6.6	0.7	-0.6	0.3	-0.1	1.2	1.1	-0.6	0.1	-0.4
hsa-miR-337	-0.2	-1.5	-2.0	-2.7	-1.0	0.9	2.8	-1.9	-1.4	-2.3
hsa-miR-33a	1.9	-1.2	-0.5	-2.3	-0.3	1.0	-2.2	-0.5	-1.1	0.0
hsa-miR-340	-1.8	-0.3	-1.5	-1.6	0.3	0.1	2.7	-2.0	0.0	-0.7
hsa-miR-361	0.7	-0.6	-0.1	0.1	-0.6	-0.2	4.8	-2.9	-0.1	-0.8
hsa-miR-377	2.2	-2.2	-0.6	0.5	-0.5	-1.5	0.1	1.0	0.2	-0.5
hsa-miR-411	-0.8	2.6	1.4	1.9	0.6	3.8	-5.3	1.7	2.4	1.4
hsa-miR-449b	0.0	0.0	-2.3	-1.3	3.1	-1.3	0.0	-3.1	-1.3	-3.0
hsa-miR-454	3.6	0.4	0.9	0.3	-3.0	-2.2	0.8	-0.2	-3.8	0.6
hsa-miR-483	-4.6	0.5	-2.1	-2.2	0.2	1.5	-3.2	-1.7	0.7	-1.9
hsa-miR-498	0.0	5.5	0.0	3.3	-0.7	0.0	-1.0	2.3	-0.3	2.0
hsa-miR-519e	0.0	0.5	0.0	1.5	-1.4	0.0	-1.5	1.7	1.4	0.0
hsa-miR-545	1.3	2.2	0.7	1.0	1.5	0.8	-1.3	0.3	2.9	0.4

Supplementary Table S5. Number of individual miRNAs, isomiRs, and hairpins detected as expressed above the minimum threshold of 10 tmm in every sample of at least one tissue. Orphan isomiRs are isomiRs that are expressed in the absence of a canonical miRNA. For this analysis we have used the adaptor trimming method to match against human hairpins.

Tissue	canonical miRNAs	isomiRs	orphan isomiRs (from hairpins)	hairpins
Brain[a]	153	1137	145 (51)	184
Heart	104	392	62 (28)	120
Kidney	123	426	50 (28)	122
Liver	103	354	63 (31)	134
Lung	78	289	53 (28)	106
Ovary	110	367	52 (29)	126
Placenta[a]	123	870	74 (33)	146
Spleen	92	354	51 (27)	111
Testes	127	445	50 (21)	137
Thymus	125	518	74 (37)	146
**TOTAL**	**242**	**2050**	**215 (107)**	**273**

[a]These tissues had RNA sourced from only one individual, and therefore isomiR number in these tissues will be overrepresented.

Supplementary Table S6. (a) List of the 47 human hairpins whose 5p arm may undergo processing through the ac-pre-miRNA pathway in at least one of the ten adult tissues sequenced. (b) Each hairpin was also examined for relative 5p expression in mutant zebra fish embryos with catalytically inactive AGO2 (see text for details). The ratio of 5p expression in the mutant/wild-type is given for each miRNA. WT means wild-type. MT means mutant. The candidates in (a) not present in (b) were not expressed above background in the WT zebra fish embryos.

(a)

**Hairpin**	**Hairpin (cont)**	**Hairpin (cont)**	**Hairpin (cont)**	**Hairpin (cont)**
hsa-let-7b	hsa-miR-146a	hsa-miR-191	hsa-miR-26b	hsa-miR-497
hsa-let-7c	hsa-miR-146b	hsa-miR-192	hsa-miR-30a	hsa-miR-509-3
hsa-let-7e	hsa-miR-150	hsa-miR-195	hsa-miR-30d	hsa-miR-510
hsa-let-7g	hsa-miR-151	hsa-miR-199b	hsa-miR-30e	hsa-miR-660
hsa-let-7i	hsa-miR-15a	hsa-miR-202	hsa-miR-31	hsa-miR-891a
hsa-miR-100	hsa-miR-15b	hsa-miR-204	hsa-miR-34a	hsa-miR-93
hsa-miR-106b	hsa-miR-17	hsa-miR-205	hsa-miR-424	hsa-miR-99a
hsa-miR-122	hsa-miR-185	hsa-miR-20a	hsa-miR-425	
hsa-miR-139	hsa-miR-186	hsa-miR-20b	hsa-miR-451	
hsa-miR-145	hsa-miR-18a	hsa-miR-21	hsa-miR-484	

(b)

**Hairpin**	**AGO2-MT/ AGO2-WT (SRP002411)**	
	canonical	isomiRs
hsa-let-7b	0	0
hsa-miR-122	0	0.04
hsa-miR-15b	0	0.02
hsa-miR-18a	0	0.02
hsa-miR-205	0	0.02
hsa-miR-20a	0.02	0.01
hsa-miR-20b	0.15	0.02
hsa-miR-30a	0	0.12
hsa-miR-30d	0.33	0.06
hsa-miR-30e	0.08	0.94
hsa-miR-34a	0	0.18
hsa-miR-451	0	0
hsa-miR-93	0	0

Supplementary Table S7. Number and proportion of isomiRs that are present in polysome extractions.

IsomiR Type	Number in Category	HeLa Polysome miRNA-seq
Canonical miRNAs	104	34 (32.7%)
Start site ismoRs	32	7 (21.9%)
End site isomiRs	243	66 (27.2%)
Substitution only isomiRs	50	0 (0.0%)
Shifted isomiRs	23	6 (26.1%)
Other mixed type isomiRs	122	9 (7.4%)
**TOTAL**	**574**	**122 (21.3%)**

Supplementary Table S8. List of synthetic RNA duplex sequences used in this study. Forward and Reverse RNA oligos were pre-hybridized by Integrated DNA Technologies.

**Name**	**Sequence (5' to 3'), and IDT modification codes**
Biotin-17-5p-F	/5Phos/rCrArA rArGrU rGrCrU rUrArC rArGrU rGrCrA rGrGrU rArG/3Bio/
Biotin-17-5p-R	/5Phos/rArCrC rUrGrC rArCrU rGrUrA rArGrC rArCrU rUrArG rArG
Biotin-10a-F	/5Phos/rUrArCrCrCrUrGrUrArGrArUrCrCrGrArArUrUrUrGrUrG/3Bio/
Biotin-10a-R	/5Phos/rCrArArArUrUrGrGrGrArUrCrUrArCrArGrGrGrCrArArG
Biotin-10a-iso-F	/5Phos/rArCrCrCrUrGrUrArGrArUrCrCrGrArArUrUrUrGrUrGrU/3Bio/
Biotin-10a-iso-R	/5Phos/rArCrArArArUrUrGrGrGrArUrCrUrArCrArGrGrArUrArG
Biotin-10b-F	/5Phos/rUrArCrCrCrUrGrUrArGrArArCrCrGrArArUrUrUrGrUrG/3Bio/
Biotin-10b-R	/5Phos/rCrArArArUrUrGrGrGrUrUrCrUrArCrArGrGrGrCrArArG
Biotin-10b-iso-F	/5Phos/rArCrCrCrUrGrUrArGrArArCrCrGrArArUrUrUrGrUrGrU/3Bio/
Biotin-10b-iso-R	/5Phos/rArCrArArArUrUrGrGrGrUrUrCrUrArCrArGrGrArUrArG
