# Supplementary material for: MicroRNAs and their isomiRs function cooperatively to target common biological pathways
Source: Genome Biol. 2011 Dec 30;12(12):R126. doi: 10.1186/gb-2011-12-12-r126 (PMC3334621; doi:10.1186/gb-2011-12-12-r126)
Supplement: Additional file 2 — miRNA-MATE source code and user manual. [file gb-2011-12-12-r126-S2.ZIP › miRNA-MATE.usermanual.pdf]

miRNA-MATE user manual

Version 1.0
January 2011
Contact:

miRNA-mate@expressiongenomics.org
Institute for Molecular Bioscience
The University of Queensland
St Lucia, QLD, 4072

License:

This software is copyright 2011 by the Queensland Centre for Medical Genomics. All rights reserved. This License is limited to, and you may use the Software solely for, your own internal and non-commercial use for academic and research purposes. Without limiting the foregoing, you may not use the Software as part of, or in any way in connection with the production, marketing, sale or support of any commercial product or service or for any governmental purposes. For commercial or governmental use, please contact licensing@qcmg.org.

In any work or product derived from the use of this Software, proper attribution of the authors as the source of the software or data must be made. The following URL should be cited:

http://grimmond.imb.uq.edu.au/miRNA-MATE/

This package is distributed in the hope that it will be useful, but WITHOUT ANY WARRANTY; without even the implied warranty of MERCHANTABILITY or FITNESS FOR A PARTICULAR PURPOSE.

Applied Biosystems Software components distributed with this package (**Mapreads**) carry their own license agreements, located at the following URLs:

http://solidsoftwaretools.com/gf/project/mapreads/

We thank and acknowledge the contributions of the developers of the above package, as well as Applied Biosystems for making the package available with the miRNA-MATE system.

Table of Content

The miRNA-MATE pipeline 4

- Introduction 4
- Availability 6
- Requirements 7

Installation instructions (miRNA-MATE) 7

Testing miRNA-MATE 9

Configuration 9

- Configuration options 11

Description of output files 15

Creating custom miRNA libraries 16

- Preprocessing the miRBase download files 16
- Finishing the hairpin libraries for mapping 17
- Condensing the mature miRNAs by mapping distance and family relationship 18
- Create a temporary library of the mature miRNAs 18
- Split the miRBase sequences by length and matching them to the temporary library 19
- Cluster all the miRNAs that multimap and assign them to miRNA families 19
- Finishing the mature miRNA libraries for matching 20

Description of modules 21

The miRNA-MATE pipeline

Introduction

miRNA-MATE is a package designed specifically for mapping and summarizing miRNA (and isomiR) data from SOLiD sequencing data sets. MicroRNAs can be particularly challenging to map because of the limited information content in a 22nt molecule. Given that we have to allow errors when mapping, and that miRNAs are very small, it is difficult to align a miRNA tag to a genome unambiguously.

To get around this, we create a library of known miRNAs to match against. The generally accepted reference repository for miRNAs is miRBase (http://www.miRBase.org). Even when considering only known miRNAs, there are significant problems with multi-mapping, because miRNAs are often very closely related. For example, miR-19a differs from miR-19b by only a single nucleotide. Until sequencing error becomes negligible, we are unlikely to be able to distinguish these miRNAs from each other. The solution that we have adopted is to collapse the miRNAs into families (which include known family members, but also miRNAs from different families that are likely to mis-map). miRNA-MATE comes with a custom library generated from miRBase 16. Alternatively, a new custom library can be made and we give instructions for doing so at the end of this manual.

miRNA-MATE can be run in either of two modes.

Mode 1: Alignment against mirBase custom library only

The first mode maps SOLiD sequencing reads against a custom library designed from miRBase (Figure 1). Unaligned tags are trimmed and re-aligned against the custom library. The resulting output is a count of miRNAs found in the sequencing run.

Mode 2: Alignment against mirBase custom library and identification of isomer usage

In the second mode, following alignment against the custom library designed from miRBase, SOLiD reads are translated into base space and analyzed for evidence of sequence variants from the canonical mirBase miRNA (Figure 1). Sequence variants can be single base substitutions, offset from the miRNA start position or length variation. The isomiR usage is summarized in form of sequence logos (Figure 2).

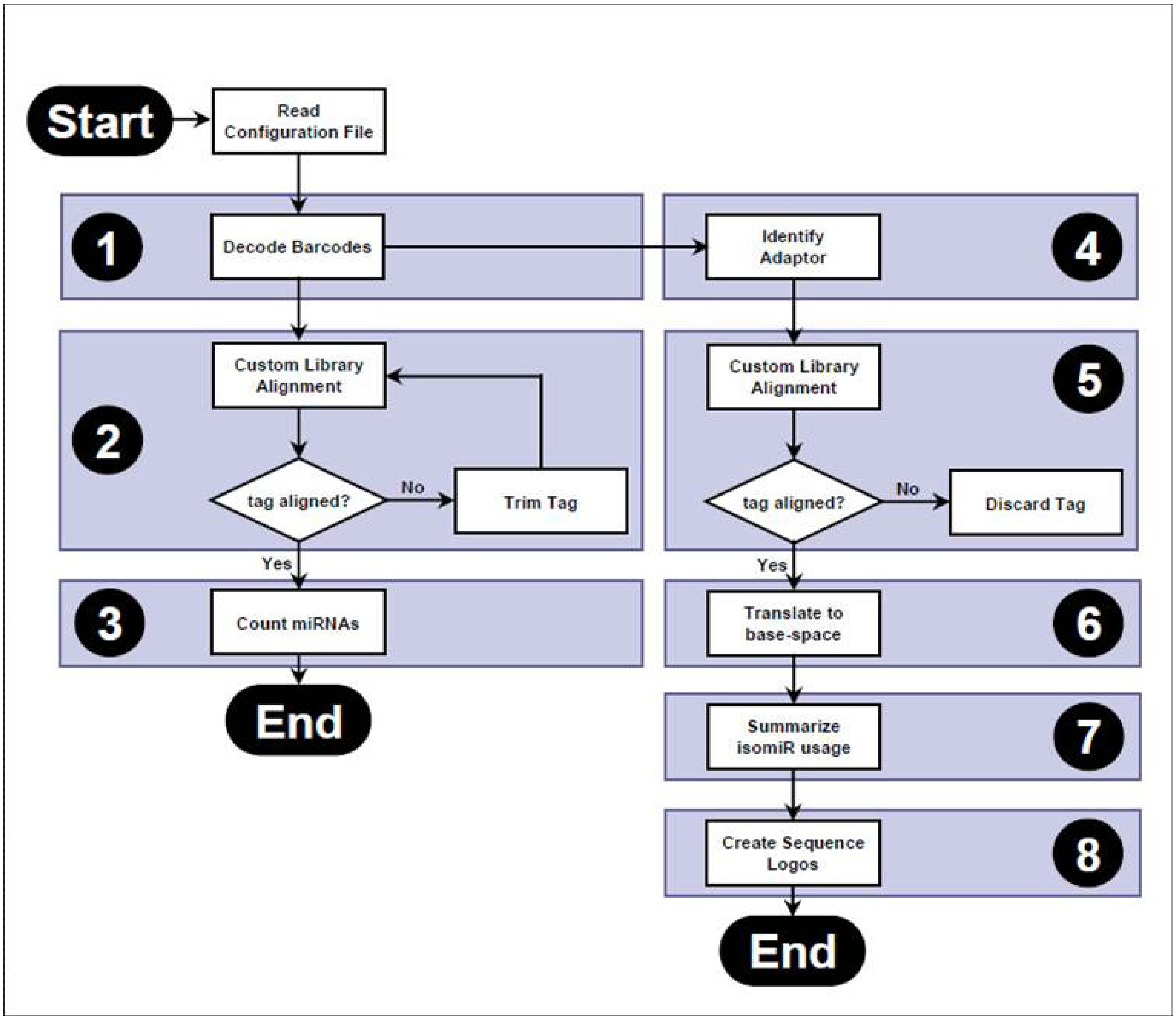

Figure 1. Outline of the miRNA-Mate pipeline. The pipeline can be run in two modes. In the first mode, only steps 1 – 3 are performed. This mode maps SOLiD sequence reads to a custom library generated from miRBase. The output contains counts for each miRNA sequence. In the second mode, the additional steps 4 – 8 are run. After alignment to the custom library, sequence reads are translated into base space and searched for evidence of isomiR usage, such as single base substitutions, miRNA start offset or length variation.

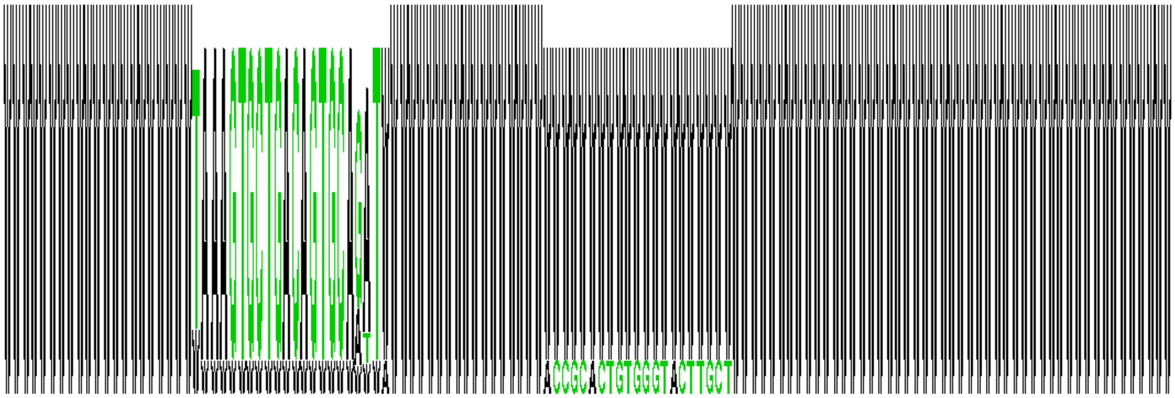

Figure 2. Example of a sequence logo produced by miRNA-MATE. If the pipeline is run in mode 2 and the data are analyzed for evidence of isomiRs, sequence logos are generated. The 'consensus' sequence of the mature miRNA and the star sequence can easily be identified. The diagram shows the full length of the hairpin. Alternatively, only the mature miRNA sequence can be represented as a sequence logo. 'W' indicates that positions were padded to make the full length hairpin sequence.

The output from miRNA-MATE includes a list of miRNAs (by their miRBase ID) encountered in the sample, the number of reads that support the canonical miRNA as represented in miRBase and the number of reads that support isomiRs, such as length, start or sequence isomiRs (Table 1).

Table 1. Example of the output summary file produced by miRNA-MATE.

#miRNA	TOTAL	CANONICAL	ISOMIR	diff_length	diff_start	diff_sequence
hsa-let-7b	7	0	7	7	7	6
hsa-let-7c	7	0	7	7	7	4
hsa-let-7d	2	0	2	2	2	0
hsa-let-7e	2	0	2	2	2	0
hsa-let-7g	6	0	6	6	6	4
hsa-let-7i	2	0	2	2	2	0

Availability

All source code, documentation, and associated files described in this manual are freely available for download from http://grimmond.imb.uq.edu.au/miRNA-MATE/

Requirements

This pipeline is written in perl and requires that you have version 5.8.8 of perl or later. It is designed to run in a unix environment, with a PBS queue manager, although running the scripts using PBS is not required.

Required perl modules are (available in CPAN):

- Parallel::ForkManager
- Path::Class
- Object::InsideOut
- Devel::StackTrace
- Class::Data::Inheritable

The alignment section of this pipeline is dependant upon the ***mapreads*** tool. This tool and its installation instructions are available from:

http://solidsoftwaretools.com/gf/project/mapreads/ (requires registration, which is free).

Additionally, the package ***weblogo*** is required and can be downloaded here:

http://weblogo.berkeley.edu/

Finally, you will need a library against which to map. The software comes with a custom library generated from miRBase version 16. Future versions of miRBase can be made into libraries using the steps outlined in section 'Creating custom miRNA libraries'. All data for the libraries can be downloaded from miRBase here:

http://www.mirbase.org/ftp.shtml

Installation instructions (miRNA-MATE)

The instructions given below in courier font are examples of the commands needed to carry out the installation. miRNA-MATE source is downloaded as a single gzipped tar file.

1. Move the tarball to the destination directory, navigate to your chosen directory and decompress miRNA-MATE.

```
mv miRNA-MATE.tar.gz /home/software/
cd /home/software/
tar xzvf miRNA-MATE.tar.gz
cd miRNA-MATE/
```

2. To install this package type the following sequence of commands:

```
perl Makefile.PL
make
make install
```

The final "`make install`" command may require that you be root or that you have permission to use the `sudo` command.

If you want to install it locally, append your location to PREFIX on the first command. eg.

```
perl Makefile.PL PREFIX=/yourDir
```

3. Move ***f2m.pl*** to the directory where ***mapreads*** is installed

```
e.g. mv /yourDir/miRNA-MATE/bin/f2m.pl /yourMapreadsDir/
```

Note: If installed properly, there should be four executable files and one schema directory:

```
f2m.pl  map  mapreads  mask_schemas_mapreads.pl  schemas
```

Note: The perl script ***f2m.pl*** requires Path::Class, which you can put in the mapreads directory if you didn't install it globally. The script ***mask_schemas_mapreads.pl*** is also in the miRNA-Mate package /bin directory and can be copied to the mapreads directory.

4. Make sure your perl path is set and includes the miRNA-MATE lib directory:

```
export PERL5LIB=/yourDir/miRNA-MATE/lib/perl5/site_perl/5.8.8/
```

5. Make sure ***weblogo*** is installed and accessible, otherwise the final logo images can’t be created.

miRNA-MATE should now be ready to test.

Testing miRNA-MATE

To ensure your installation is functioning correctly, we have provided test data and comparison results within the miRNA-MATE package in the /test/ directory.

The test data directory includes:

- miRNA_mate_input.csfasta
- miRNA_mate_example.conf
- miRBasev16.hairpin.hsa.padded.cat
- miRBasev16.hairpin.hsa.padded.index
- results/ (the directory of results, for information about the files in here see the section ‘Description of output files’).

INPORTANT: Edit the configuration file so that it refers to the appropriate directories that you have set up on your system. You can then run the pipeline using the following command:

```
nohup perl [path]/mirna_mate.pl –c miRNA_mate_example.conf &
```

Where `[path]` is the full path to miRNA-Mate.pl

Testing will run miRNA-MATE in approximately 15 minutes assuming your PBS queue is empty. Once the run has completed, the results should be compared to those provided in the /results/ folder. In particular, the .log file should be inspected for any errors, and the .freq files should be compared for any differences.

If there are no differences (other than time stamps and directory names) between your results and the packaged results then the installation is working correctly.

Configuration

The configuration file is a text file containing all the required parameters to run miRNA-MATE. In this file, directory listings must end with a “/”, there must be no other punctuation at the end of the lines, and there should be no empty lines in this file. An example of the configuration file is given on the next page (Figure 3).

```
exp_name=S0014_20080526_2_PolysomeAndAtlas_microRNA_Atlas
output_dir=/data/cxu/MiRNA/scripts.Jun18/test/
raw_F3=/data/cxu/post_mapping_data/S0014_20080526_2_PolysomeAndAtlas_microRNA_Atlas_F3.csfasta
raw_BC=/data/cxu/post_mapping_data/S0014_20080526_2_PolysomeAndAtlas_microRNA_Atlas_R3.csfasta
mask=11111111111111111111111111111111111
max_multimatch=5
raw_tag_length=35
Adaptor_start=33020
Adaptor_start_mismatch=1
max_adaptor_mismatch=5
name_Adaptor=Adaptor_BC1,Adaptor_BC2,Adaptor_BC3,Adaptor_BC4,Adaptor_BC5,Adaptor_BC6,Adaptor_BC7,
Adaptor_BC8,Adaptor_BC9,Adaptor_BC10
Adaptor_BC1=33020103031311231200032032222031220201003000312
Adaptor_BC2=33020103031311231200111122222031220201003000312
Adaptor_BC3=33020103031311231220200022222031220201003000312
Adaptor_BC4=33020103031311231230323122222031220201003000312
Adaptor_BC5=33020103031311231231013132222031220201003000312
Adaptor_BC6=33020103031311231201130012222031220201003000312
Adaptor_BC7=33020103031311231201221022222031220201003000312
Adaptor_BC8=33020103031311231221302222222031220201003000312
Adaptor_BC9=33020103031311231202020022222031220201003000312
Adaptor_BC10=33020103031311231202103312222031220201003000312
name_BC=BC1,BC2,BC3,BC4,BC5,BC6,BC7,BC8,BC9,BC10
BC_length=5
BC1=G00032
BC2=G00111
BC3=G20200
BC4=G30323
BC5=G31013
BC6=G01130
BC7=G01221
BC8=G21302
BC9=G02020
BC10=G02103
max_miRNA_length=29
miRNA_freq_filter=100
miRNA_start_offset=10
miRNA_end_offset=10
w_seq_length=50
recursive_map=true
recursive_map_para=30.1.0,29.1.0,28.1.0,27.1.0,26.1.0,25.1.0,24.1.0,23.1.0,22.1.0,21.1.0,20.1.0,1
9.1.0,18.1.0,17.1.0
once_map_para=29.2.1,28.2.1,27.2.1,26.2.1,25.2.1,24.2.1,23.2.1,22.2.1,21.2.1,20.2.1,19.2.1,18.2.1
,17.2.1
expect_strand=+
run_seqlogo=/data/weblogo/seqlogo -f "input" -F GIF -h 10 -w 30 -k 0 -o "output" -c
f2m=/data/cxu/mapping_strategy/matching/f2m.pl
mapreads=/data/cxu/mapping_strategy/matching/mapreads
script_editing=/data/cxu/mapping_strategy/matching/editing.pl
recursive_library=/data/cxu/MiRNA/library/discovery_miRNA_library.padded.cat
recursive_library_index=/data/cxu/MiRNA/library/discovery_miRNA_library.padded.index
once_library=/data/cxu/MiRNA/library/human_miRNA_editing_library.padded.cat
once_library_index=/data/cxu/MiRNA/library/human_miRNA_editing_library.padded.index
```

Figure 3. Example of a miRNA-MATE configuration file.

Configuration options

[Standard Parameters]

exp_name = S0014_20080526_2_PolysomeAndAtlas_microRNA_Atlas

Set the experiment name with this parameter.

output_dir = /data/MiRNA/20080526_output/

Specify the full path of the output directory. This directory must exist prior to running the pipeline.

raw_F3 = /data/cxu/post_mapping_data/S0014_20080526_2_PolysomeAndAtlas_microRNA_Atlas_F3.csfasta

Specify the full path of the .csfasta file to be mapped.

raw_tag_length = 35

This parameter defines the longest length of the tags contained in the .csfasta file.

mask = 11111111111111111111111111111111111

This setting allows you to ignore particular bases in the tag when computing the number of mismatches. 1 = consider this base, 0 = do not consider this base. The length of the mask should equal the length of the longest tags.

max_multimatch = 5

Defines the maximum number of positions to be reported for multi-mapping tags. The higher this number, the more disk space is required to store the data and the slower the program will run. Recommended size for most applications is 10.

expect_strand = +

This defines the strandedness of the data. For example, libraries made with the SREK protocol or other direct ligation protocols will have tags that are sequenced in the sense (+) strand relative to the expressed gene. Libraries made with the SQRL protocol will have tags that are sequenced in the antisense (-) relative to the expressed gene.

[Barcode Parameters]

name_BC = BC1,BC2,BC3,BC4,BC5,BC6,BC7,BC8,BC9,BC10

This parameter lists all barcode names. All related barcode sequence must be listed on this configure file when more than one barcode are used for whole samples. See below example.

```
BC1=G00032
BC2=G00111
BC3=G20200
BC4=G30323
BC5=G31013
BC6=G01130
BC7=G01221
BC8=G21302
BC9=G02020
BC10=G02103
```

BC_length = 5

Indicate the length of barcode sequence.

raw_BC = /data/cxu/post_mapping_data/S0014_20080526_2_PolysomeAndAtlas_microRNA_Atlas_R3.csfasta

This file lists each tag's barcode sequence which must belong to the barcodes listed in the configure file. Eg. BC1 … BC10. It is not necessary to list this parameter if all tags are from same barcode.

[Adaptor Parameters]

name_Adaptor = Adaptor_BC1,Adaptor_BC2,Adaptor_BC3,Adaptor_BC4,Adaptor_BC5,Adaptor_BC6,Adaptor_BC7,Adaptor_BC8,Adaptor_BC9,Adaptor_BC10

This parameter lists all adaptor names. Each adaptor name must follow format: "Adaptor"_[BC], the [BC] must be consistent with the barcode names listed in "name_BC". All related adaptor sequences must be listed in this configure file. See below example:

```
Adaptor_BC1=330201030313112312000320322220312202010030003l2
Adaptor_BC2=33020103031311231200111122222031220201003000312
Adaptor_BC3=33020103031311231220200022222031220201003000312
Adaptor_BC4=33020103031311231230323122222031220201003000312
Adaptor_BC5=33020103031311231231013132222031220201003000312
Adaptor_BC6=33020103031311231201130012222031220201003000312
Adaptor_BC7=33020103031311231201221022222031220201003000312
Adaptor_BC8=33020103031311231221302222222031220201003000312
Adaptor_BC9=33020103031311231202020022222031220201003000312
Adaptor_BC10=33020103031311231202103312222031220201003000312
```

Adaptor_start = 33020

The pipeline will strip the adaptor sequences from the raw tags and then map each tag. To improve the speed for finding adaptor sequences, the program first searches a short sequence identical to the start of the adaptor sequences. If a match is found, then the rest of the adapter sequence is compared.

```
Adaptor_start_mismatch = 1
max_adaptor_mismatch = 5
```

Specify these parameters to set the maximum mismatch value for searching the adaptor start sequence and the maximum mismatch value for searching the whole adaptor sequence.

[Mapping Parameters]

```
once_map_para = 21.2.1,20.2.1
```

These parameters define the lengths at which matching will occur for tags after the adapter sequences have been stripped off, the number of mismatches permissible between the tag and the reference sequence, and whether or not to treat valid adjacent errors as a single mismatch. These are dot-separated parameters with the format of **[length]**.**[mismatches]**.**[valid_adjacent]**. **[length**] defines the length of the tag to match at, **[mismatches]** defines the number of mismatches allowed, and **[valid_adjacent]** is set to 1 if valid adjacent errors are to be treated as a single mismatch, or 0 if they are not.

In the above example, tags of length 21 will be mapped using the parameters 2 mismatches and treating valid adjacent mismatches as a single mismatch.

NOTE: Mapping schemas must be available to do the mapping at the specified length and number of mismatches, or else the pipeline will fail. In this example, the schemas required are:

```
schema_21_2_adj
schema_20_2_adj
```

Mapping schemas are available from http://solidsoftwaretools.com/

```
f2m = /data/cxu/mapping_strategy/matching/f2m.pl
mapreads = /data/cxu/mapping_strategy/matching/mapreads
```

These parameters define the full paths of the scripts/programs required to run miRNA-MATE.

```
once_library = /data/MiRNA/library/human_miRNA_editing_library.padded.cat
once_library_index =
/data/MiRNA/library/human_miRNA_editing_library.padded.index
```

These parameters define the miRNA libraries and their associated index files that are used in miRNA-MATE.

[Recursive Mapping Parameters]

```
recursive_map = true
```

```
recursive_map_para =
30.1.0,29.1.0,28.1.0,27.1.0,26.1.0,25.1.0,24.1.0,23.1.0,22.1.0,21.1.0,20.1
.0,19.1.0,18.1.0,17.1.0
recursive_library = /data/MiRNA/library/discovery_miRNA_library.padded.cat
recursive_library_index =
/data/MiRNA/library/discovery_miRNA_library.padded.index
```

If you set recursive mapping to **true**, you must list recursive mapping parameters in the "recursive_map_para" parameter. These parameters define the lengths at which matching will occur recursively. The naming convention is the same as for the "once_map_para". When recursively mapping, a tag which fails to map at length N is then truncated and remapped at a smaller length. The amount of truncation is specified in the above parameters.

NOTE: miRNA-MATE libraries for mature (canonical) sequences will need to be created with the appropriate amount of mismatches incorporated, see section 'Creating custom miRNA libraries'.

[Parameters for analyzing isomiRs]

These parameters only apply if miRNA-MATE is run in Mode 2 where miRNA sequences are searched for evidence of isomiRs, such as miRNAs that differ by a single base or whose start position is offset compared to the canonical miRNA.

```
script_editing = /data/cxu/mapping_strategy/matching/editing.pl
```

This parameter lists the full path to the script that is used to retrieve the base sequence and information regarding the number of mismatches when comparing to the reference file.

```
run_seqlogo = /data/weblogo/seqlogo -f "input" -F GIF -h 10 -w 30 -k 0 -o
"output" -c
```

This parameter not only lists the full path to the script, but also allows the user to set multiple input parameters. The "input" and "output" files will be replaced automatically when running this script as part of miRNA-Mate.

```
max_miRNA_length = 29
```

After adapter sequences have been stripped, tags shorter than this length are treated as miRNA sequences.

```
miRNA_freq_filter = 100
```

miRNA sequences are only reported if they appear more than this number of times.

```
miRNA_start_offset = 10
miRNA_end_offset = 10
```

We append letter “N” to each end of the reference sequences in the miRNA library. These two parameters indicate how many “N”s are appended on each side of the miRNA reference sequence.

```
w_seq_length = 50
```

For visualization purposes, we add the letter “w” on both sides of high frequency miRNA sequences whose adaptor sequences have been stripped. This procedure makes these sequences the same length as non-stripped sequences. The length can be specified here.

Description of output files

For all files listed below, the string <expname> refers to the experiment name provided by the **exp_name** configuration parameter, <BC> refers to one of the names given for the bar codes using the **name_BC** parameter, <run_type> is either ‘recursive’ or ‘once’, <direction> is either ‘positive’ (tag matched in the expected orientation) and ‘negative’ (tag matched on the reverse strand to the direction provided in the library file). <seqLength> refers to the length of the input sequence and will vary according to the optional recursive strategy employed. <libName> is the name of the library files. This is used to prefix some raw mapping output files. <miRNA> is the name of the matched miRNA (or miRNA family) found in the dataset and listed in the provided library files.

- **<expname>.<BC>.log –** The log file for the run. This file contains important information about the status of a run, and should help diagnose any potential errors.
- **<expname>.<BC>.<runtype>.SIM.<direction>.freq –** A ‘frequency’ file, containing the frequency of occurrence for each miRNA (or miRNA family) in the data set.
- **<expname>.<BC>.<runtype>.SIM.<direction>.summary –** A summary file of the miRNAs, isomiRs, length and start differences for all identified miRNAs in the input data set.
- **<expname>.<BC>.<runtype>.SIM.<direction>.PreSummary[.sorted] –** An intermediate file used to create the ‘.summary’ file described above.
- **<expname>.<BC>.<runtype>.SIM.<direction>.sorted.ID –** An intermediate file used to create the frequency file described above.
- **<expname>.<BC>.<runtype>.SIM.<direction>.start_end.sorted –** Another intermediate file used when creating the summary and frequency files listed above.
- **<expname>.<BC>.mersN.<csfasta>** - If the parameter recursive_map is set to ‘true’, these files contain the csfasta (colour-space fasta) files of the input data, truncated to the specified length. Only tags which do not map at the longer length are included in subsequent (shorter) length csfasta files.
- **<libname>.<expname>.<BC>.<seqLength>.csfasta.ma.N.M.*** - Raw mapping output files, including STDOUT, STDERR, flag files, shell scripts etc. N=tag length, and M=number of mismatches. These files are ‘collated’ to produce summarized mapping output files, such as the .collated file listed below.
- **<expname>.<BC>.<runtype>.collated –** The mapping results for either recursive or ‘once’ mapping runs.

- **<expname>.<BC>.<runtype>.<miRNA>** - FASTA file containing aligned sequences for the specified miRNA, used to create the weblogo.
- **<expname>.<BC>.<runtype>.<miRNA>.gif** – Weblogo image file for this miRNA
- **<expname>.<BC>.NT<N>** - Csfasta file containing all adaptor trimmed sequences of ‘N’ length.
- **<expname>.<BC>.<runtype>.SIM.<direction>** - These files list the single match (SIM) tags in both the positive and negative directions.
- **< expname>.<BC>.<runtype>.SIM.<direction>.bc[sorted]** – These files provide details about the exact location of matched tags on miRNA hairpins (the offset, relative to the canonical miRNA location), as well as the tag name, sequence, reference sequence, and number of mismatches. The ‘.sorted’ file is sorted by reference miRNA sequence.

Creating custom miRNA libraries

While miRNA-MATE comes with a custom library for mapping miRNAs, new custom libraries can be made following the steps outlined here. The process requires access to the UCSC genome browser and perl scripts provided as part of the miRNA-MATE package.

Preprocessing the miRBase download files

1. Download miRNA sequences from miRBase. The files ‘mature.fa’ and ‘hairpin.fa’ are required.

2. Reformat the files using the script ***01_make_one_line_fasta.pl*** provided in the miRNA-MATE package. This script takes multi-line fasta files and converts the file to a one-line fasta format (sequence spans a single line with no line break). This step makes is easier to work with the files, but is not important for the mature sequences which are typically so small that they don’t span multiple lines anyway.

```
./01_make_one_line_fasta.pl -f miRBasev15/hairpin.fa -o
hairpin.fa.one_line
./01_make_one_line_fasta.pl -f miRBasev15/mature.fa -o
mature.fa.one_line
```

3. Run the script ***02_change_U_to_T_and_remove_description.pl*** provided in the miRNA-MATE package. This script strips the header information and converts the U (uracil) to T (thymine). **This step is important.** If you fail to convert the Us to Ts, the mapping fill fail later.

```
./02_change_U_to_T_and_remove_description.pl -f hairpin.fa.one_line -o
hairpin.fasta
```

```
./02_change_U_to_T_and_remove_description.pl -f mature.fa.one_line -o
mature.fasta
```

4. Select the species for mapping using the script ***00_filter_species.pl*** provided as part of the miRNA-MATE package. This final script selects the species that may be of particular interest. This is best done for the hairpin library, but not for the mature miRNA library. Having all species in the mature library allows one to determine the specificity of matching, but also allows the possibility of miRNA discovery leveraging the knowledge of other species. This can be quite difficult to do in colourspace otherwise, due to the mapping specificity issues discussed above. The following example takes the hairpin.fasta file and extracts only the human (hsa) hairpins into an output file called hairpin.fasta.hsa.

```
./00_filter_species.pl -f hairpin.fasta -o hairpin.fasta.hsa -s hsa
```

This is all the preprocessing that needs to happen before continuing with the library construction. Hairpin libraries are very straightforward to finish from this point (next section), but mature miRNA libraries need a little more processing (the section after next).

Finishing the hairpin libraries for mapping

There are three scripts required to finish off the hairpin library ready for matching.

1. The first script pads the ends of the hairpin miRNA with a string of 10 N's. This prevents short tags mapping across the boundaries of hairpins when they are all concatenated for the final library.

```
./03_pad_mature_miRNAs.pl -f hairpin.fasta.hsa -o
hairpin.fasta.hsa.padded
```

2. The second script makes an index file so that miRNA-MATE can deconvolve the matching positions and return a miRNA ID. **Important**: you must use the padded file to create the index, or the coordinates will be wrong.

```
./04_make_index.pl -f hairpin.fasta.hsa.padded -o
hairpin.fasta.hsa.padded.index
```

3. The final script concatenates the miRNAs ready for matching with ***mapreads***.

```
./05_concatenate_sequences.pl -f hairpin.fasta.hsa.padded -o
hairpin.fasta.hsa.padded.cat -h miRBasev15_human_hairpins
```

Congratulations, you have just built a hairpin library and index suitable for use with miRNA-MATE! You will need both the *.index and *.cat files to pass to miRNA-MATE in its configuration file.

Condensing the mature miRNAs by mapping distance and family relationship

Making mature miRNA libraries is a little more involved because the sequences are small and miRNAs that cross-map as a single entity need to be grouped. Additionally, it is beneficial to include miRNAs from other species (to determine the level of background mapping noise versus miRNA expression), however these additional miRNAs may well be identical to miRNAs in the species you are interested in, and could therefore cause multi-mapping. The process has the following basic steps:

- create a temporary library of the mature miRNAs
- split the miRBase sequences by length, and match them to the temporary library (ie. themselves) at a given number of mismatches
- collect and cluster all the miRNAs that multi-map and assign them to miRNA families
- collect all related miRNAs and assign them to miRNA families
- create the *.index and *.cat files as above for hairpin miRNAs
- make separate and custom indices for the recursive matching and the isomiR matching

Note: Because the miRNAs are mapped at a specific number of mismatches, the library you create here will only be valid for matching with that same number of mismatches. Hence, different libraries will be required each time matching is performed using a different mismatch number. For example, a 1MMVA library (allows 0-1 colour space errors, counting valid adjacent errors as one error) should not be used as a reference when matching is performed allowing 2MMVA (2 colour space errors, counting valid adjacent errors as one error). Allowing fewer mismatches would be fine.

Create a temporary library of the mature miRNAs

This process is identical to the process described above for finishing the hairpin libraries.

```
./03_pad_mature_miRNAs.pl -f mature.fasta -o mature.fasta.padded

./04_make_index.pl -f mature.fasta.padded -o mature.fasta.padded.index

./05_concatenate_sequences.pl -f mature.fasta.padded -o
mature.fasta.padded.cat -h temp_miRNA_library_miRBasev15
```

Split the miRBase sequences by length and matching them to the temporary library

Mapreads will only match at one given length at a time, but miRNAs in miRBase range from 16-35 nucleotides. In order to match them all, the miRNAs in the miRBase files need to be separated by length. The following scripts will split the miRBase input files and convert them to colour space.

1. This script takes the preprocessed mature sequences and splits them by length, outputting a series of files called split_miRBase.[$length].

   ```
   ./06_split_by_length.pl -f mature.fasta
   ```

2. The next script takes the output of the above script, and converts the nucleotide sequences to colourspace tags (prepends a T and converts to colourspace). There are no arguments for this script (it searches the current working directory).

   ```
   ./07_convert_to_colourspace_with_initial_T.pl
   ```

3. The next script takes these .csfasta files and submits the matching jobs to the queue. You need to specify the temporary mature miRNA library (created above, eg. mature.fasta.padded.cat), as well as the mismatch parameters.

   ```
   ./08_match_miRs.pl -l mature.fasta.padded.cat -p
   /data/matching/mapreads -s /data/matching/schemas/ -m 2 -a 1
   ```

   This matching step does not take very long, since there is a tiny number of samples mapping against a tiny search space. At most, this script will take about 2 minutes to finish at which point all of the matching results should be concatenated into a single file. The directory can then be cleaned up by removing the temporary files.

   ```
   cat *.2.1 >mapping_results.2MMVA
   rm split_miRbase.*
   ```

Cluster all the miRNAs that multi-map and assign them to miRNA families

The next step is to identify those miRNAs that are cross-mapping and thus have the potential to allow mismapping to occur. miRNAs are assigned to miRNA families based on their match positions and names.

1. Identify clusters of miRNAs with similar matching positions. This script decodes the matching positions using the specified index file and clusters those miRNAs together that have similar matching positions, one line per miRNA cluster.

```
./09_make_miRNA_clusters.pl -f mature.fasta.padded.index -m
mapping_results.2MMVA -o mapping_results.2MMVA.clusters
```

2. The next script then takes these clusters and makes .fasta sequences from them, removing duplicate sequences as well as those that are fully contained within another sequence.

```
./10_create_new_fasta.pl -f mature.fasta -m
mapping_results.2MMVA.clusters -o mapping_results.2MMVA.clusters.fasta
```

3. miRNAs with similar names (e.g. has-mir-27a and has-mir-27b) generally have similar sequences and these ‘families’ might be missed when mapping at a particular threshold, especially if the miRNA contain insertions or deletions. Experience shows, however, that shadow matching to these family members will occur in large enough data sets. To remove this shadow matching, members of the same family that might be more distantly related are collapsed. This next script performs pattern matching on the miRNA name to retrieve all family members and output the complete miRNA library.

```
./11_final_cluster_by_family.pl -f mapping_results.2MMVA.clusters.fasta
-o miRBasev15_mature_miRNA_library.2MMVA
```

Finishing the mature miRNA libraries for matching

Like the temporary matching libraries and the hairpin libraries, the same three basic scripts need to be run on the complete miRNA library.

```
./03_pad_mature_miRNAs.pl -f miRBasev15_mature_miRNA_library.2MMVA -o
miRBasev15_mature_miRNA_library.2MMVA.padded

./04_make_index.pl -f miRBasev15_mature_miRNA_library.2MMVA.padded -o
miRBasev15_mature_miRNA_library.2MMVA.padded.index

./05_concatenate_sequences.pl -f
miRBasev15_mature_miRNA_library.2MMVA.padded -o
miRBasev15_mature_miRNA_library.2MMVA.padded.cat -h
miRBasev15_mature_library_all_miRNAs
```

However, the long IDs in the index file will cause havoc with miRNA-MATE unless these are condensed. Two more indices need to be generated specifically for miRNA-MATE. The first will be for recursive matching, and the second will be for isomiR "once only" mapping.

```
./12_reannotate_index_file.pl -f
miRBasev15_mature_miRNA_library.2MMVA.padded.index -p hsa
```

The only difference between the two indices is that the recursive index has duplicate names, whereas the isomiR index has unique names (Figure 4).

```
::::::::::::::
miRBasev15_mature_miRNA_library.2MMVA.padded.index.isomiR.hsa
::::::::::::::
miRNA-family__miR169_1  0       40
miRNA-family__miR169_2  42      82
miRNA-family__miR169_3  84      124
miRNA-family__miR169_4  126     166
miRNA-family__miR169_5  168     209

::::::::::::::
miRBasev15_mature_miRNA_library.2MMVA.padded.index.recursive.hsa
::::::::::::::
miRNA-family__miR169    0       40
miRNA-family__miR169    42      82
miRNA-family__miR169    84      124
miRNA-family__miR169    126     166
miRNA-family__miR169    168     209
```

Figure 4. The top panel shows an example of an index file for isomiR 'once only' mapping, while the bottom panel shows an example of an index file for a recursive mapping. Note the duplicate names in the bottom panel used for recursive mapping.

This allows the recursive matching to add together all the counts for the family as a single number, and prevents errors in miRNA-MATE image generation scripts from complaining because there is no data.

And that's it! Congratulations, you should now have successfully made a mature miRNA library suitable for use with miRNA-MATE.

Description of modules

For module descriptions, please use the embedded PerlDoc, by typing:

```
perldoc <ModuleName.pm>
```
