# Supplementary material for: MicroRNAs and their isomiRs function cooperatively to target common biological pathways
Source: Genome Biol. 2011 Dec 30;12(12):R126. doi: 10.1186/gb-2011-12-12-r126 (PMC3334621; doi:10.1186/gb-2011-12-12-r126)
Supplement: Additional file 4 — Tab-delimited text file of the raw counts of isomers detected in the libraries created in this study. [file gb-2011-12-12-r126-S4.PDF]

Supplementary File 2. Nomenclature for isomiR description.

Goals of the nomenclature:

1. To create a standard way to describe variants of known miRNA sequences derived from deep sequencing data
2. To create a machine-parseable nomenclature to concisely convey all of the sequence information regarding an individual isomiR that is completely described within the isomiR name. ie. given the hairpin sequence, one should be able to derive the sequence of the isomiR from its name.

- isomiRs are described in relation to a hairpin precursor, and the first part of the isomiR name will be the which arm of this hairpin it has derived from
- "Star" nomenclature is removed, and replaced with either -5p, -3p, or –amb, depending on the localization of the expressed miRNA in relation to the hairpin
- Canonical sequence name is separated from the variant description by a pipe ("|")
- Nucleotide 0 is the first nucleotide of the hairpin sequence as defined in miRBase
- All nucleotide numbers are given in relation to the hairpin sequence as defined in miRBase
- All variations take the form [affected nucleotides].[type].[affected bases]
- All variations are listed from 5' to 3'
- Multiple variations are separated by a semi-colon (";");
- "sub" indicates a substitution, ">" indicates the specific substitution in the affected bases
- "del" indicates a deletion
- "ins" indicates an insertion
- "ext" indicates an extension beyond the miRBase annotated hairpin
- "_" indicates a range of nucleotides

<u>Examples</u>:

1. hsa-miR-182-5p|{hsa-miR-182}|22_45|
   This is the canonical miR-182 sequence

   hsa-miR-182-5p|{isomiR}|22_43|
   This is an isomiR of miR-182 that is shorter by 2nt but has no substitutions.

2. hsa-miR-143-3p|{hsa-miR-143}|60_80|
   This is the canonical miR-143 sequence

   hsa-miR-143-3p|{isomiR}|60_80|sub.77.G>A;sub.79.T>C
   This is an isomiR of miR-143 that has the same length as the canonical, but has two substitutions at position 77 and 79 of the hairpin.

3. hsa-miR-29a-3p|{hsa-miR-29a}|41_62|
   This is the canonical miR-29a sequence

   hsa-miR-29a-3p|{isomiR}|41_64|ext.64.N
   This is an isomiR of miR-29a that is 2 nt longer than the canonical miR and 1 nt beyond the miRBase annotated hairpin
